# Intramolecular carbolithiation of *N*-allyl-ynamides: an efficient entry to 1,4-dihydropyridines and pyridines – application to a formal synthesis of sarizotan

**DOI:** 10.3762/bjoc.8.250

**Published:** 2012-12-21

**Authors:** Wafa Gati, Mohamed M Rammah, Mohamed B Rammah, Gwilherm Evano

**Affiliations:** 1 Institut Lavoisier de Versailles, UMR CNRS 8180, Université de Versailles Saint-Quentin-en-Yvelines, 45, avenue des Etats-Unis, 78035 Versailles Cedex, France; 2 Laboratoire de Chimie Organique Hétérocyclique LCOH/LCOHSNR-LR11ES39, Département de Chimie, Faculté des Sciences de Monastir, Université de Monastir, avenue de l’environnement, 5019 Monastir, Tunisia,; 3 Laboratoire de Chimie Organique, Service de Chimie et PhysicoChimie Organiques, Université Libre de Bruxelles, Avenue F. D. Roosevelt 50, CP160/06, 1050 Brussels, Belgium

**Keywords:** carbolithiation, carbometallation, dihydropyridines, organolithium reagents, pyridines, sarizotan, ynamides

## Abstract

We have developed a general synthesis of polysubstituted 1,4-dihydropyridines and pyridines based on a highly regioselective lithiation/6-endo-dig intramolecular carbolithiation from readily available *N*-allyl-ynamides. This reaction, which has been successfully applied to the formal synthesis of the anti-dyskinesia agent sarizotan, further extends the use of ynamides in organic synthesis and further demonstrates the synthetic efficiency of carbometallation reactions.

## Introduction

Since the discovery of the carbometallation reaction by Ziegler and Bähr in 1928 [1], this reaction has evolved as a most powerful tool to construct carbon–carbon bonds. An ever increasing number of organometallic species have been shown over the years to be suitable reagents for the carbometallation of various carbon–carbon multiple bonds. Lithium, copper, zinc, magnesium, zirconium, titanium, palladium and other metals are suitable for this transformation and considerable progress has recently been made in this area. Among these systems, the carbometallation of alkynes constitutes a most efficient entry to polysubstituted alkenes, provided that both the regioselectivity and the stereoselectivity can be controlled [2–5]. In this context, an efficient strategy to control these selectivity issues is the incorporation of a heteroatom directly attached to the triple bond, which can dramatically affect both the regio- and stereochemical outcomes due to the polarization of the triple bond and/or the formation of chelation-stabilized vinylmetal species. The carbometallation of O-, N-, P-, S-, and Si-substituted alkynes has indeed been quite extensively studied and shown to provide most efficient entries to polysubstituted, stereodefined heteroatom-substituted alkenes and has been implemented in remarkably elegant processes [6]. Intramolecular versions are especially attractive and provide most useful entries to highly substituted carbo- and heterocycles.

**Scheme 1:**
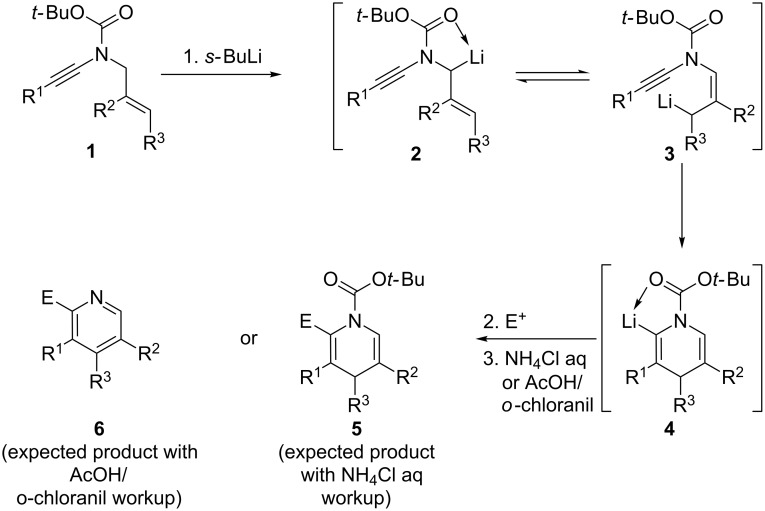
Strategy for the synthesis of (1,4-dihydro)pyridines by deprotonation/intramolecular carbolithiation.

Based on our recent interest in the chemistry of ynamides [7–18] and inspired by recent reports from Meyer and Cossy [19–21], Marek [22–24] and Lam [25–26] on their carbopalladation, carbocupration and carbozincation, respectively, we decided to study the intramolecular carbolithiation of ynamides, which may provide an interesting entry to highly functionalized 1,4-dihydropyridines [27–29] and pyridines [30–33], most useful building blocks in organic synthesis and medicinal chemistry as well. Our strategy is summarized in Scheme 1 and is based on the following assumptions: According to the remarkable work of the Beak group on the α-lithiation of Boc-protected amines [34–38], *N*-allyl-ynamides **1** should be readily deprotonated to afford a transient chelation-stabilized allyllithium **2** and, provided that a metallotropic equilibrium exists between this intermediate and the less-stable allyllithium **3**, an intramolecular carbometallation may then occur to yield a chelation-stabilized vinyllithium **4** and drive the overall process to the formation of the heterocyclic ring system. Further reaction with an electrophile followed by aqueous workup or hydrolysis under acidic and oxidative conditions would then afford the highly substituted dihydropyridine or pyridine derivatives **5** and **6**, respectively. While there were no examples of such exclusive anionic 6-endo-dig cyclizations reported to the best of our knowledge [39], we felt that the presence of the chelating group on the nitrogen may allow such a selective process and a clean formation of the (dihydro)pyridine ring system. We have indeed demonstrated the efficiency of this strategy [40] and now report in this manuscript a full account on this work as well as the application of our pyridine synthesis to a formal synthesis of the anti-dyskinesia agent sarizotan.

## Results and Discussion

### Feasibility of the deprotonation/intramolecular carbolithiation

**Scheme 2:**
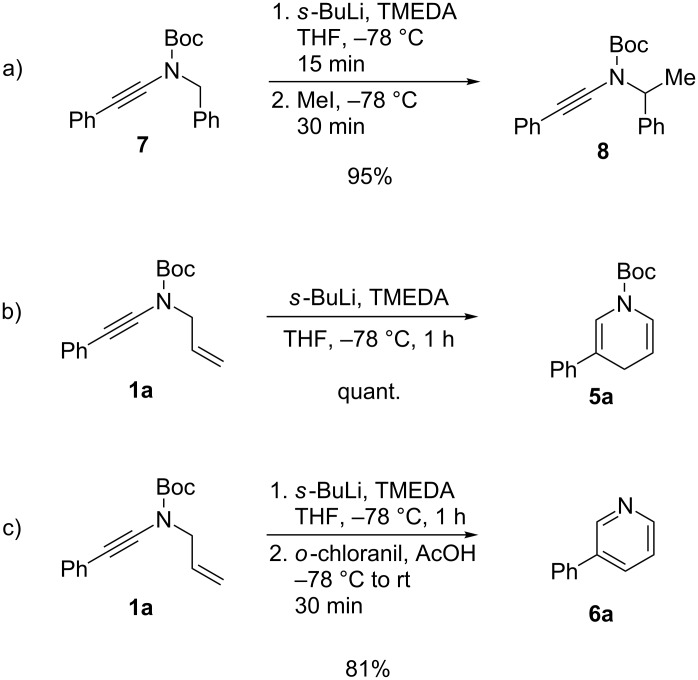
Feasibility of the deprotonation/intramolecular carbolithiation.

To first evaluate the compatibility of the ynamide moiety with the lithiation step and address potential problems associated with competitive carbolithiation of the activated alkyne, we first reacted *N*-benzyl-ynamide **7** with one equivalent of *sec*-butyllithium and tetramethylethylenediamine (TMEDA) in THF at −78 °C for 15 minutes, followed by the addition of methyl iodide. The corresponding *N*-phenylethyl-ynamide **8** was obtained in nearly quantitative yield, therefore demonstrating the compatibility of the ynamide group with the deprotonation step (Scheme 2a), although longer reaction times before the addition of methyl iodide resulted in much lower yields and extensive degradation: The intramolecular carbolithiation of *N*-allyl-ynamides to 1,4-dihydropyridines might therefore be possible, provided that the overall reaction rate is not too slow. To further test this hypothesis, *N*-allyl-ynamide **1a** was reacted under similar reaction conditions and to our delight, a smooth cyclization occurred, the expected 1,4-dihydropyridine **5a** being virtually formed as virtually the sole product (Scheme 2b), a clean reaction being a strict requirement for the development of a general route to 1,4-dihydropyridines devoided of an electron-withdrawing group at C-3, due to the instability of these acid- and oxygen-sensitive molecules. In situ conversion of the intermediate dihydropyridine to the corresponding pyridine by replacing the saturated aqueous ammonium chloride solution, used for the workup, by a combination of acetic acid and *o*-chloranil [41] was equally successful and provided the expected pyridine **6a** in 81% yield (Scheme 2c).

### Synthesis of the starting *N*-allyl-ynamides

**Scheme 3:**
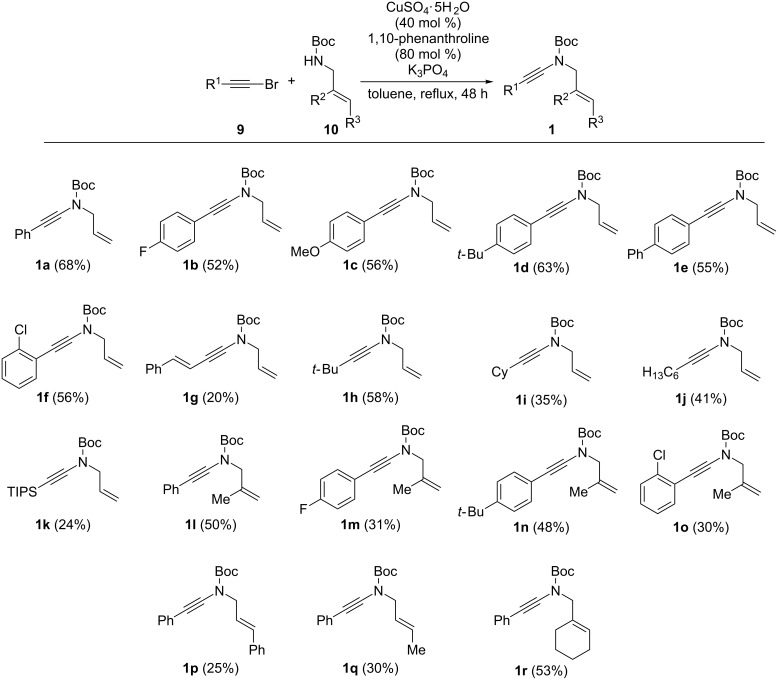
Synthesis of the starting *N*-allyl-ynamides.

Before moving to the evaluation of the scope and limitations of this intramolecular carbolithiation, we had to prepare a set of ynamides possessing representative substituents on both the ynamide and allyl groups. Among all the methods evaluated, Hsung’s second-generation synthesis based on the copper-mediated cross-coupling between bromoalkynes **9** and nitrogen nucleophiles [42] turned out to be the most convenient one, the use of terminal alkynes [43], *gem*-dibromoalkenes [7], potassium alkynyltrifluoroborates [8] or copper acetylides [12] being less efficient when bulky *N*-Boc-allylamines **10** were used as nucleophiles. By using a slightly modified Hsung’s procedure, a series of *N*-allyl-ynamides **1** could be readily prepared in acceptable yields using a combination of copper(II) sulfate pentahydrate (40 mol %) and 1,10-phenanthroline (80 mol %) with potassium phosphate in refluxing toluene, the major side reaction observed in all cases being the competitive dimerization of the starting bromoalkynes (Scheme 3).

### Intramolecular carbolithiation of *N*-allyl-ynamides to 1,4-dihydropyridines and pyridines: scope and limitations

**Scheme 4:**
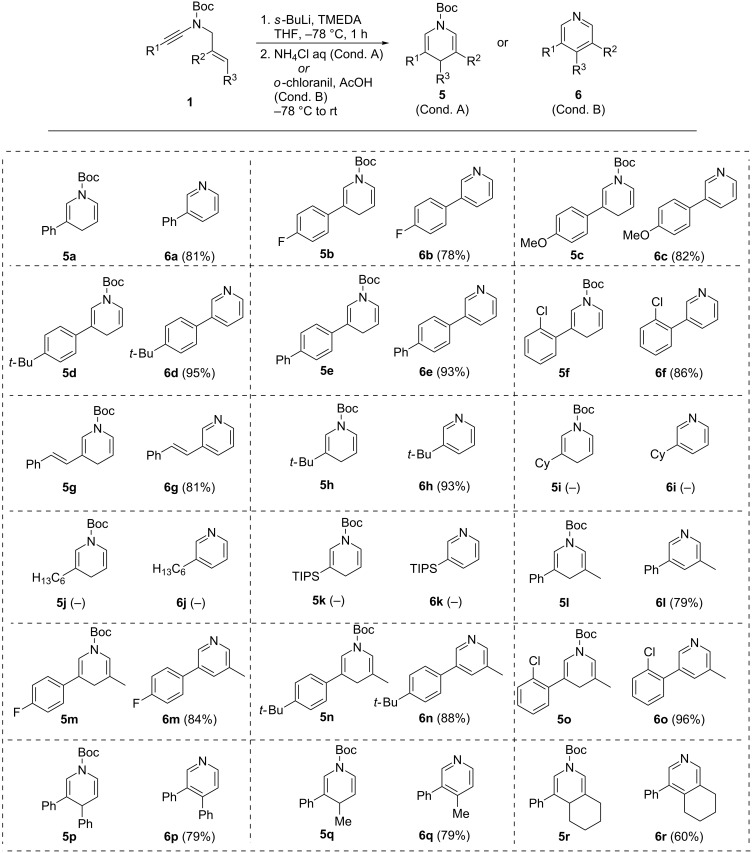
Intramolecular carbolithiation of *N*-allyl-ynamides to 1,4-dihydropyridines and pyridines.

With this set of ynamides in hand, we next evaluated their cyclization to the corresponding 1,4-dihydropyridines **5** or pyridines **6** using the lithiation/intramolecular carbolithiation sequence. All *N*-allyl-ynamides **1** shown in Scheme 3 were therefore treated with *s*-butyllithium and TMEDA in THF at −78 °C for one hour followed by the addition of an aqueous saturated solution of ammonium chloride (Scheme 4, conditions A) or acetic acid and *o*-chloranil (Scheme 4, conditions B), yielding the corresponding 1,4-dihydropyridines **5** or pyridines **6**, respectively. Results from these studies are collected in Scheme 4, yields being indicated only in the pyridine series due to the high sensitivity of the 1,4-dihydropyridine derivatives, which were obtained in virtually pure form in crude reaction mixtures. As evidenced with these results, 3-aryl-1,4-dihydropyridines and pyridines (**5**/**6a**–**f** and **l**–**r**) are readily obtained from the corresponding aryl-substituted *N*-allyl-ynamides regardless of the substitution pattern or the electronic properties of the aromatic ring. The reaction is also efficient in the case of an alkenyl group (**5**/**6g**) and the presence of an alkyl group was, as expected, more problematic due to competitive propargylic lithiation. Indeed, while a *tert*-butyl group was well tolerated (**5**/**6h**), the presence of secondary or primary alkyl chains such as cyclohexyl (**5**/**6i**) or *n*-hexyl (**5**/**6j**) groups did not afford the cyclized products, which could also not be obtained either when starting from a TIPS-protected primary ynamide (**5**/**6k**), the silicon protecting group being readily cleaved under the reaction conditions. The influence of the substitution pattern of the allyl moiety was next carefully examined and substitution at the β-, γ-positions, or both, was well tolerated, yielding the 3,5-disubstituted- (**5**/**6l**–**o**), 3,4-disubstituted- (**5**/**6p**–**q**) and 3,4,5-trisubstituted- (**5**/**6r**) 1,4-dihydropyridines and pyridines in good yields, respectively, compounds that are rather challenging to obtain using other synthetic routes. In addition, the reaction can be performed on a gram-scale with similar efficiency. This was indeed briefly evaluated for the anionic cyclization of ynamide **1a** and the exact same yield of the corresponding pyridine **6a** (81%) was obtained on a gram scale.

**Scheme 5:**
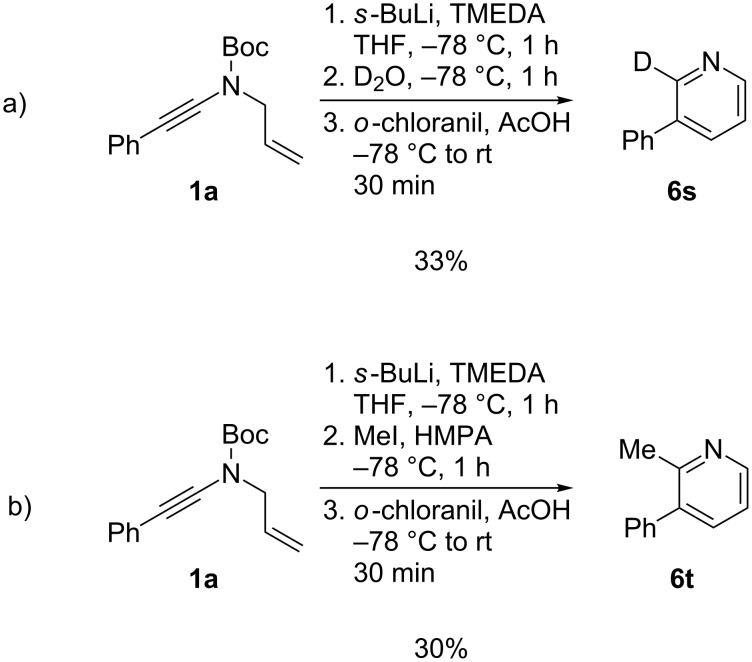
2,3-Disubstituted pyridines by trapping of the intermediate metallated 1,4-dihydropyridine.

With a chelation-stabilized vinyllithium being formed after the anionic 6-endo-dig cyclization, we next considered the possibility of trapping this vinyllithium with an electrophile, which might allow the introduction of an additional C-2 substituent. *N*-Allyl-ynamide **1a** was therefore cyclized under our standard conditions and then treated with deuterated water (Scheme 5a) or methyl iodide (Scheme 5b) before the acidic and oxidative workup. While the desired 2,3-disubstituted **6s** and **6t** were indeed formed under these conditions, they could only be isolated in modest yields (30–33%), even in the presence of additional HMPA, which might may constitute the major limitation of our process. Other attempts involving electrophiles such as acid chlorides and allyl bromide or transmetallation with zinc chloride and Negishi cross-coupling were unsuccessful, which does confirm that our deprotonation/carbometallation sequence is not suitable for the preparation of C-2-substituted (1,4-dihydro)pyridines.

### Application to a formal synthesis of sarizotan

**Scheme 6:**
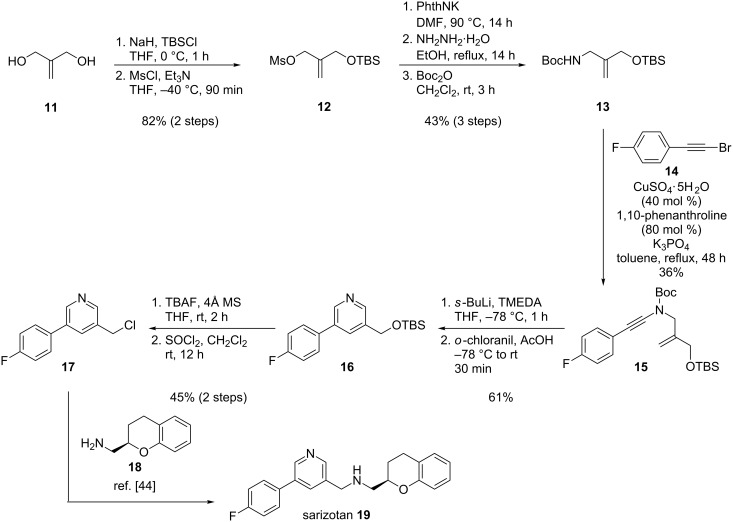
Formal synthesis of the anti-dyskinesia agent, 5-HT_1A_ receptor agonist, dopamine D_2_ receptor ligand sarizotan.

To further probe the synthetic utility of our pyridine synthesis, we next envisioned its use for the synthesis of the 3,5-disubstituted pyridine core of the antidyskinetic drug, 5-HT_1A_ receptor agonist, dopamine D_2_ receptor ligand sarizotan **19** (Scheme 6). The pyridinyl-chroman sarizotan (also called EMD-128130) was originally developed by Merck KGaA in the late 1990’s [44] and was found to be a dual selective 5-HT_1A_ receptor agonist and D_2_ receptor antagonist displaying a strong efficacy in the reduction of dyskinesia resulting from long-term antiparkinsonian treatment with levodopa [45–50]. Although its development was stopped by Merck KGaA in 2006 after analysis of data from Phase III clinical trials failed to confirm its efficiency [51], sarizotan is still under intense investigation [52–54] and was recently licensed to Newron Pharmaceuticals for further testing in new indications [55].

Motivated by the high potential of sarizotan and by the clear lack of structure-activity relationship studies on the pyridine core of this bioactive compound [56], we designed an efficient and modular synthesis of the disubstituted pyridine core of sarizotan that should enable the preparation of sarizotan analogues with different substitution on the pyridine ring. This synthesis is shown in Scheme 6 and starts from commercially available 2-methylene-1,3-propanediol (**11**). Mono-protection of this diol as a TBS ether and activation of the remaining alcohol as a mesylate according to previously reported procedures [57–58], gave allylic mesylate **12**, which was next reacted with potassium phthalimide in DMF at 90 °C, affording the corresponding *N*-allylphthalimide. Deprotection of the phthalimide by hydrazinolysis followed by protection of the resulting primary amine as a *tert*-butyl-carbamate gave the Boc-protected amine **13** required for the synthesis of the substrate of the anionic cyclization. Indeed, this amine **13** was engaged in a copper-catalyzed cross-coupling with 1-(bromoethynyl)-4-fluorobenzene (**14**) and gave the corresponding ynamide **15** in a modest, unoptimized 36% yield. This set the stage for the key lithiation/intramolecular carbolithiation/oxidation step for the formation of the pyridine ring. To our delight, treatment of **14** under our optimized conditions (treatment with *s*-butyllithium and tetramethylethylenediamine in THF at −78 °C for one hour followed by addition of acetic acid and *o*-chloranil) smoothly promoted the anionic 6-endo-dig cyclization to the 3,5-disubstituted pyridine **16**, which was isolated in 61% yield. Further deprotection of the primary alcohol with TBAF followed by chlorination with thionyl chloride finally gave the desired chloromethylpyridine **17**, an intermediate used for the preparation of sarizotan **19** at Merck KGaA by coupling of this pyridine fragment **17** with aminomethylchroman **18** [44].

## Conclusion

The lithiation/isomerization/6-endo-dig intramolecular carbolithiation sequence from readily available *N*-allyl-ynamides provides an efficient entry to highly substituted 1,4-dihydropyridines and pyridines and has been successfully implemented in a formal synthesis of the anti-dyskinesia agent sarizotan. This new addition to the field of carbometallation reactions extends the chemistry of ynamides and should be useful in heterocyclic and medicinal chemistry as well. Further studies to extend this process to other heteroatom-substituted alkynes and to develop an asymmetric version of our 1,4-dihydropyridines synthesis are in progress and will be reported in due timecourse.

## Supporting Information

Experimental details and copies of NMR spectra for all new compounds.

File 1 Experimental.

File 2 Copies of ^1^H and ^13^C NMR spectra for new compounds.
