# Supplementary material for: Intramolecular carbolithiation of N-allyl-ynamides: an efficient entry to 1,4-dihydropyridines and pyridines – application to a formal synthesis of sarizotan
Source: Beilstein J Org Chem. 2012 Dec 21;8:2214–22. doi: 10.3762/bjoc.8.250 (PMC3557957; doi:10.3762/bjoc.8.250)
Supplement: File 1 — Experimental. [file Beilstein_J_Org_Chem-08-2214-s001.pdf]

Supporting Information File 1

for

Intramolecular carbolithiation of *N*-allyl-ynamides: an efficient entry to 1,4-dihydropyridines and pyridines – application to a formal synthesis of sarizotan

Wafa Gati,[1,2] Mohamed M. Rammah,[2] Mohamed B. Rammah,[2] and Gwilherm Evano*,[3]

Address : [1]Institut Lavoisier de Versailles, UMR CNRS 8180, Université de Versailles Saint-Quentin-en-Yvelines, 45, avenue des Etats-Unis, 78035 Versailles Cedex, France, [2]Laboratoire de Chimie Organique Hétérocyclique, Département de Chimie, Faculté des Sciences de Monastir, Université de Monastir, avenue de l'environnement, 5019 Monastir, Tunisia, and [3]Laboratoire de Chimie Organique, Service de Chimie et PhysicoChimie Organiques, Université Libre de Bruxelles, Avenue F. D. Roosevelt 50, CP160/06, 1050 Brussels, Belgium

Email: Gwilherm Evano - gevano@ulb.ac.be

* Corresponding author

Experimental

General Information

All reactions were carried out in oven-dried glassware under an argon atmosphere employing standard techniques in handling air-sensitive materials.

All solvents were reagent grade. THF and toluene were freshly distilled from sodium/benzophenone under argon and degassed immediately prior to use. Dichloromethane, DMF and *N*,*N*,*N'*,*N'*-tetramethylethylenediamine were freshly distilled over calcium hydride.

Finely powdered potassium phosphate tribasic (Alfa Aesar) was used for copper-mediated coupling reactions. All other reagents were used as supplied.

Reactions were magnetically stirred and monitored by thin-layer chromatography using Merck-Kieselgel $60F_{254}$ plates. Flash chromatography was performed with silica gel 60 (particle size 35–70 μm) supplied by SDS. Yields refer to chromatographically and spectroscopically pure compounds, unless otherwise noted.

^{1}H NMR spectra were recorded by using an internal deuterium lock at ambient temperature on a Bruker 300 MHz spectrometer. Internal reference of δ_H 7.26 was used for $CDCl_3$. Data are presented as follows: chemical shift (in ppm on the δ scale relative to δ_{TMS} = 0), multiplicity (s = singlet, d = doublet, t = triplet, q = quadruplet, quint. = quintuplet, sext. = sextuplet, m = multiplet, br. = broad, app. = apparent), coupling constant (*J*/Hz) and integration. Resonances that are either partially or fully obscured are denoted obscured (obs.). ^{13}C NMR spectra were recorded at 75 MHz. Internal reference of δ_C 77.16 was used for $CDCl_3$. ^{19}F NMR spectra were recorded at 282 MHz.
Infrared spectra were recorded on a Nicolet iS 10 (SMART iTR diamond ATR) spectrophotometer. Mass spectra were obtained on a Waters XevoQTof spectrometer.
The synthesis and characterization of compounds **1a**-**h**, **1j**, **1l**-**r**, **5a**-**r**, **6a**-**r** have already been reported in our preliminary communication [1].

***N*-(1-Phenylethyl)-*N*-(*tert*-butoxycarbonyl)phenylethynylamine (8).** To a solution of *N*-benzyl-*N*-(*tert*-butoxycarbonyl)phenylethynylamine **7** [2] (1.0 mmol) in freshly distilled THF (2 mL) was added *N*,*N*,*N'*,*N'*-tetramethylethylenediamine (TMEDA, 170 μL, 1.1 mmol). The resulting mixture was cooled to −78 °C and treated with *s*-butyllithium (1.4 M in cyclohexane, 825 μL, 1.2 mmol) dropwise. The resulting dark red solution was stirred at −78 °C for 15 minutes and methyl iodide (185 μL, 2.0 mmol) was added. The reaction mixture was stirred at −78 °C for 30 minutes, quenched with an aqueous saturated solution of ammonium chloride and warmed to room temperature. The aqueous layer was extracted with EtOAc and the combined organic layers were washed twice with brine, dried over $MgSO_4$, filtered and concentrated to yield ynamide **8** as a pale yellow oil (302 mg, 0.94 mmol, 95%). ^{1}H NMR (300 MHz, $CDCl_3$): δ 7.35 (d, J = 7.3 Hz, 2H), 7.42-7.29 (m, 8H), 5.30-5046 (br. m, 1H), 1.74 (d, J = 7.1 Hz, 3H), 1.55 (s, 9H) ; ^{13}C NMR (75 MHz, $CDCl_3$): δ 153.8, 141.3, 130.5, 128.5, 128.3, 128.0, 127.0, 126.9, 124.0, 82.6, 81.7, 73.2 (br.), 55.4 (br.), 27.8, 19.1 (br.); IR (ATR) ν_{max} 2973, 2917, 2240, 1744, 1688, 1500, 1442, 1385 cm^{-1}; EIHRMS *m/z* calcd for $C_{21}H_{23}NaNO_2$ $[M + Na]^+$ 344.1623, found 344.1626.

***N*-Allyl-*N*-(*tert*-butoxycarbonyl)cyclohexylethynylamine (1i).** To a solution of the (bromoethynyl)cyclohexane (1.9 g, 10.0 mmol) in dry toluene (50 mL), were sequentially added *N*-Boc-allylamine (1.7 g, 11.0 mmol), potassium phosphate (5.1 g, 24.0 mmol), copper(II) sulfate pentahydrate (1.0 g, 4.0 mmol) and 1,10-phenanthroline (1.45 g, 8.0 mmol). The reaction mixture was heated under reflux for 2 days, cooled to room temperature, filtered over a plug of silica gel (washed with EtOAc), and concentrated. The crude residue was purified by flash column chromatography over silica gel (petroleum ether) to yield the desired ynamide **1i** as a colorless oil (922 mg, 3.5 mmol, 35%). ^{1}H NMR (300 MHz, $CDCl_3$): δ 5.84 (ddt, J = 17.0, 10.1 and 6.0 Hz, 1H), 5.25-5.16 (m, 2H), 3.95 (d, J = 6.0 Hz, 2H), 2.51-2.44 (m, 1H), 1.74-1.63 (m, 4H), 1.53-1.24 (m, 15H); ^{13}C NMR (75 MHz, $CDCl_3$): δ 154.7, 132.5, 117.9, 82.1, 75.0 (br.), 73.6 (br.), 52.0 (br.), 33.2, 29.0, 28.3, 26.2, 24.8; IR (ATR) ν_{max} 2974, 2931, 2852, 2260, 1716, 1456, 1397 cm^{-1}; ESIHRMS *m/z* calcd for $C_{16}H_{25}NNaO_2$ $[M + Na]^+$ 286.1783, found 286.1783.

***N*-Allyl-*N*-(*tert*-butoxycarbonyl)(triisopropylsilyl)ethynylamine (1k).** To a solution of the (bromoethynyl)triisopropylsilane (2.6 g, 10.0 mmol) in dry toluene (50 mL), were sequentially added *N*-Boc-allylamine (1.7 g, 11.0 mmol), potassium phosphate (5.1 g, 24.0 mmol), copper(II) sulfate pentahydrate (1.0 g, 4.0 mmol) and 1,10-phenanthroline (1.45 g, 8.0 mmol). The reaction mixture was heated under reflux for 2 days, cooled to room temperature, filtered over a plug of silica gel (washed with EtOAc), and concentrated. The crude residue was purified by flash column chromatography over silica gel (petroleum ether) to yield the desired ynamide **1i** as a colorless oil (810 mg, 2.4 mmol, 24%). ^{1}H NMR (300 MHz, $CDCl_3$): δ 5.90 (ddt, *J* = 17.1, 11.3 and 6.0 Hz, 1H), 5.25-5.18 (m, 2H), 4.02 (d, *J* = 6.0 Hz, 2H), 1.50 (s, 9H), 1.08 (s, 21H); ^{13}C NMR (75 MHz, $CDCl_3$): δ 153.9, 131.8, 118.3, 97.7 (br.), 82.3, 67.8 (br.), 52.0 (br.), 28.0, 18.6, 11.3; IR (ATR) ν_{max} 2934, 2859, 2168, 1726, 1463, 1363 cm^{-1}; ESIHRMS *m/z* calcd for $C_{14}H_{28}NSi$ [M – Boc + H]$^+$ 238.1991, found 238.1990.

***N*-Phthaloyl-2-(*tert*-butyldimethylsilyloxymethyl)allylamine.** To a solution of **12** [3] (6.7 g, 24.0 mmol) in dry DMF (80 mL) was added potassium phthalimide (4.9 g, 26.4 mmol). The reaction mixture was heated to 90 °C overnight, cooled to room temperature and quenched with water. The aqueous layer was extracted with ether and the combined organic layers were washed with brine, dried over $MgSO_4$, filtered and concentrated. The crude residue was purified by flash column chromatography over silica gel (petroleum ether/EtOAc: 8/2) to give the corresponding protected allylic amine as a colorless oil (5.0 g, 15.1 mmol, 63%). ^{1}H NMR (300 MHz, $CDCl_3$): δ 7.87 (dd, *J* = 5.3 and 3.1 Hz, 2H), 7.74 (dd, *J* = 5.3 and 3.1 Hz, 2H), 5.20 (s, 1H), 5.00 (s, 1H), 4.30 (s, 2H), 4.20 (s, 2H), 0.90 (s, 9H), 0.07 (s, 6H); ^{13}C NMR (75 MHz, $CDCl_3$): δ 168.0, 142.8, 134.0, 132.1, 123.3, 111.6, 64.7, 39.6, 25.8, 18.3, −5.4; IR (ATR) ν_{max} 2953, 2928, 2855, 1773, 1713, 1389, 1114, 1085, 834, 713 cm^{-1}; EIHRMS *m/z* calcd for $C_{18}H_{26}NO_3Si$ [M + H]$^+$ 332.1682, found 332.1684.

***N*-*tert*-Butoxycarbonyl-2-(*tert*-butyldimethylsilyloxymethyl)allylamine (13).** To a solution of *N*-phthaloyl-2-(*tert*-butyldimethylsilyloxymethyl)allylamine (3.3 g, 10.0 mmol) in EtOH (55 mL) was added hydrazine hydrate (1.0 mL, 19.8 mmol). The reaction

mixture was heated under reflux overnight, cooled to room temperature and concentrated. The crude residue was dissolved in dichloromethane (8 mL) and a solution of di-*tert*-butyl dicarbonate (2.2 g, 10.0 mmol) in dichloromethane (2 mL) was added dropwise at 0 °C. The reaction mixture was allowed to warm to room temperature, stirred for 3 hours and concentrated. The crude residue was finally purified by flash chromatography over silica gel (petroleum ether/EtOAc: 7/3) to give the corresponding Boc-protected allylic amine **13** as a colorless oil (2.1 g, 6.8 mmol, 68%). ^{1}H NMR (300 MHz, $CDCl_3$): δ 5.07 (s, 1H), 5.00 (s, 1H), 4.86 (br. s, 1H), 4.11 (s, 2H), 3.70-3.71 (m, 2H), 1.41 (s, 9H), 0.88 (s, 9H), 0.04 (s, 6H); ^{13}C NMR (75 MHz, $CDCl_3$): δ 155.9, 145.4, 110.8, 79.2, 64.8, 43.1, 28.4, 25.8, 18.3, −5.5; IR (ATR) ν_{max} 3338, 3194, 2929, 2857, 1692, 1250, 1108, 775 cm^{-1}; EIHRMS *m/z* calcd for $C_{15}H_{31}NNaO_3Si$ [M + Na]$^+$ 324.1971, found 324.1964.

***N*-[2-(*tert*-Butyldimethylsilyloxymethyl)allyl]-*N*-*tert*-butoxycarbonyl-(4-fluorophenyl)ethynylamine (15).** To a solution of 1-(bromoethynyl)-4-fluoro-benzene **14** (2.0 g, 10.0 mmol) in dry toluene (50 mL), were sequentially added **13** (3.3 g, 11.0 mmol), potassium phosphate (5.1 g, 24.0 mmol), copper(II) sulfate pentahydrate (1.0 g, 4.0 mmol) and 1,10-phenanthroline (1.45 g, 8.0 mmol). The reaction mixture was heated under reflux for 2 days, cooled to room temperature, filtered over a plug of silica gel (washed with EtOAc), and concentrated. The crude residue was purified by flash chromatography over silica gel (petroleum ether) to yield the desired ynamide **15** as a pale yellow oil (1.5 g, 3.6 mmol, 36%). ^{1}H NMR (300 MHz, $CDCl_3$): δ 7.32 (app. t, *J* = 8.8 Hz, 2H), 6.97 (app. t, *J* = 9.0 Hz, 2H), 5.28 (s, 1H), 5.13 (s, 1H), 4.20 (s, 2H), 4.12 (s, 2H), 1.54 (s, 9H), 0.92 (s, 9H), 0.08 (s, 6H); ^{13}C NMR (75 MHz, $CDCl_3$): δ 161.8 (d, *J* = 246 Hz), 153.9, 143.0, 132.6, 120.0, 115.4 (d, *J* = 22 Hz), 112.4, 83.4 (br.), 82.7, 69.3 (br.), 64.0, 51.3 (br.), 28.0, 25.9, 18.4, −4.7; ^{19}F NMR (282 MHz, $CDCl_3$): δ −113.7; IR (ATR) ν_{max} 3344, 3188, 2929, 2857, 1692, 1366, 1250, 1152, 1108, 779, 775 cm^{-1}; EIHRMS *m/z* calcd for $C_{23}H_{34}FNNaO_3Si$ [M + Na]$^+$ 442.2190, found 442.2195.

3-(*tert*-Butyldimethylsilyloxymethyl)-5-(4-fluorophenyl)pyridine (16). To a solution of ynamide **15** (420 mg, 1.0 mmol) in freshly distilled THF (2 mL) was added *N*,*N*,*N'*,*N'*-

tetramethylethylenediamine (TMEDA, 170 μL, 1.1 mmol). The resulting mixture was cooled to −78 °C and treated with *s*-butyllithium (1.4 M in cyclohexane, 825 μL, 1.2 mmol) dropwise. The resulting dark red solution was stirred at −78 °C for 1 hour, treated with glacial acetic acid (2 mL) and 3,4,5,6-tetrachloro-1,2-benzoquinone (*o*-chloranil, 246 mg, 1.0 mmol), warmed to room temperature, stirred for 30 minutes and quenched with an aqueous saturated solution of ammonium chloride. The aqueous layer was extracted with EtOAc and the combined organic layers were washed twice with brine, dried over $MgSO_4$, filtered and concentrated. The crude residue was purified by flash chromatography over silica gel (petroleum ether/EtOAc: 8/2) to give the desired pyridine **16** as a pale yellow oil (194 mg, 0.61 mmol, 61%). ^{1}H NMR (300 MHz, $CDCl_3$): δ 8.70 (s, 1H), 8.53 (s, 1H), 7.86 (s, 1H), 7.54 (dd, *J* = 8.8 and 5.6 Hz, 2H), 7.19 (app. t, *J* = 8.5 Hz, 2H), 4.84 (s, 2H), 0.96 (s, 9H), 015 (s, 6H); ^{13}C NMR (75 MHz, $CDCl_3$): δ 162.9 (d, *J* = 246.5 Hz), 146.1, 145.9, 137.1, 135.7, 133.8, 132.6, 128.7 (d, *J* = 8.2 Hz), 116.1 (d, *J* = 21.5 Hz), 62.6, 27.3, 25.9, −5.3; ^{19}F NMR (282 MHz, $CDCl_3$): δ −113.9; IR (ATR) ν_{max} 2953, 2929, 2856, 1771, 1606, 1094, 815, 775 cm^{-1}; EIHRMS *m/z* calcd for $C_{18}H_{25}NOFSi$ $[M + H]^+$ 318.1689, found 318.1683.

5-(4-Fluorophenyl)-3-(hydroxymethyl)pyridine. To a solution of **16** (320 mg, 1.0 mmol) and 4 Å molecular sieves (100 mg) in dry THF (10 mL) was added dropwise at 0 °C a solution of tetra-*n*-butylammonium fluoride (1M in THF, 2.0 mL, 2.0 mmol). The resulting yellow solution was warmed to room temperature, stirred for 8 hours and quenched with an aqueous saturated solution of ammonium chloride. The aqueous layer was extracted with EtOAc, and the combined organic layers were washed twice with brine, dried over $MgSO_4$, filtered and concentrated. The crude residue was purified by flash chromatography over silica gel (EtOAc/EtOH: 8/2) to give the desired deprotected pyridinyl alcohol as a white solid (93 mg, 0.46 mmol, 46%). ^{1}H NMR (300 MHz, $CDCl_3$): δ 8.60 (s, 1H), 8.48 (s, 1H), 7.88 (s, 1H), 7.49 (dd, *J* = 8.3 and 5.4 Hz, 2H), 7.14 (app. t, *J* = 8.5 Hz, 2H), 4.79 (s, 2H), 4.25 (br. s, 1H); ^{13}C NMR (75 MHz, $CDCl_3$): δ 162.9 (d, *J* = 246.3 Hz), 146.7, 146.6, 136.9, 135.7, 133.5, 133.3, 128.7 (d, *J* = 8.2 Hz), 116.1 (d, *J* = 21.5 Hz), 62.2;^{19}F NMR (282 MHz, $CDCl_3$): δ −113.8; IR (ATR)

ν_{max} 3275, 2922, 2850, 1721, 1605, 1560, 1432, 1232, 1012, 836 cm^{-1}; EIHRMS *m/z* calcd for $C_{12}H_{11}NOF$ $[M + H]^+$ 204.0825, found 204.0825.

5-(4-Fluorophenyl)-3-(chloromethyl)pyridine (17). To a solution of 5-(4-fluorophenyl)-3-(hydroxymethyl)pyridine (45 mg, 0.22 mmol) in dichloromethane (0.4 mL) was added dropwise at 0 °C thionyl chloride (17 μL, 0.23 mmol). The resulting solution was allowed to warm to room temperature, stirred for 4 hours and quenched with water. The aqueous layer was washed with dichloromethane, and the combined organic layers were washed twice with brine, dried over $MgSO_4$, filtered and concentrated to give the corresponding analytically pure chlorinated pyridine **17** as a white solid (40 mg, 0.18 mmol, 84%), no further purification being needed. ^1H NMR (300 MHz, $CDCl_3$): δ 8.82 (br. s, 1H), 8.64 (br. s, 1H), 7.90 (s, 1H), 7.57 (dd, *J* = 8.5 and 5.2 Hz, 2H), 7.19 (app. t, *J* = 8.6 Hz, 2H), 4.67 (s, 2H); ^{13}C NMR (75 MHz, $CDCl_3$): δ 160.7 (d, *J* = 246.9 Hz), 145.7, 145.6, 134.9, 132.2, 130.9, 131.0, 126.6 (d, *J* = 8.1 Hz), 113.9 (d, *J* = 21.5 Hz), 40.7; ^{19}F NMR (282 MHz, $CDCl_3$): δ −113.5; IR (ATR) ν_{max} 2957, 2920, 2850, 1607, 1514, 1435, 1228, 884, 717 cm^{-1}; EIHRMS *m/z* calcd for $C_{12}H_{10}NFCl$ $[M + H]^+$ 222.0486, found 222.0483.

References

1. Gati, W.; Rammah, M. M.; Rammah, M. B.; Couty, F.; Evano, F. *J. Am. Chem. Soc.* **2012,** *134*, 9078–9081.
2. Istrate, F. M.; Buzas, A. K.; Jurberg, I. D.; Odabachian, Y.; Gagosz, F. *Org. Lett.* **2008,** *10*, 925–928.
3. Couladourous, E. A.; Dakanali, M.; Demadis, K. D.; Vidali, V. P. *Org. Lett.* **2009,** *11*, 4430–4433.
