# Supplementary material for: Intramolecular carbolithiation of N-allyl-ynamides: an efficient entry to 1,4-dihydropyridines and pyridines – application to a formal synthesis of sarizotan
Source: Beilstein J Org Chem. 2012 Dec 21;8:2214–22. doi: 10.3762/bjoc.8.250 (PMC3557957; doi:10.3762/bjoc.8.250)
Supplement: File 2 — Copies of 1H and 13C NMR spectra for new compounds. [file Beilstein_J_Org_Chem-08-2214-s002.pdf]

Supporting Information File 2

for

Intramolecular carbolithiation of *N*-allyl-ynamides: an efficient entry to 1,4-dihydropyridines and pyridines – application to a formal synthesis of sarizotan

Wafa Gati,[1,2] Mohamed M. Rammah,[2] Mohamed B. Rammah,[2] and Gwilherm Evano*,[3]

Address : [1]Institut Lavoisier de Versailles, UMR CNRS 8180, Université de Versailles Saint-Quentin-en-Yvelines, 45, avenue des Etats-Unis, 78035 Versailles Cedex, France, [2]Laboratoire de Chimie Organique Hétérocyclique, Département de Chimie, Faculté des Sciences de Monastir, Université de Monastir, avenue de l'environnement, 5019 Monastir, Tunisia, and [3]Laboratoire de Chimie Organique, Service de Chimie et PhysicoChimie Organiques, Université Libre de Bruxelles, Avenue F. D. Roosevelt 50, CP160/06, 1050 Brussels, Belgium

Email: Gwilherm Evano - gevano@ulb.ac.be

* Corresponding author

Copies of ^{1}H and ^{13}C NMR spectra for new compounds

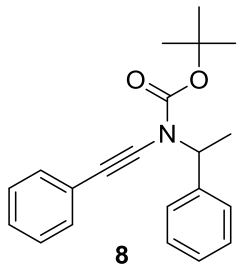

8

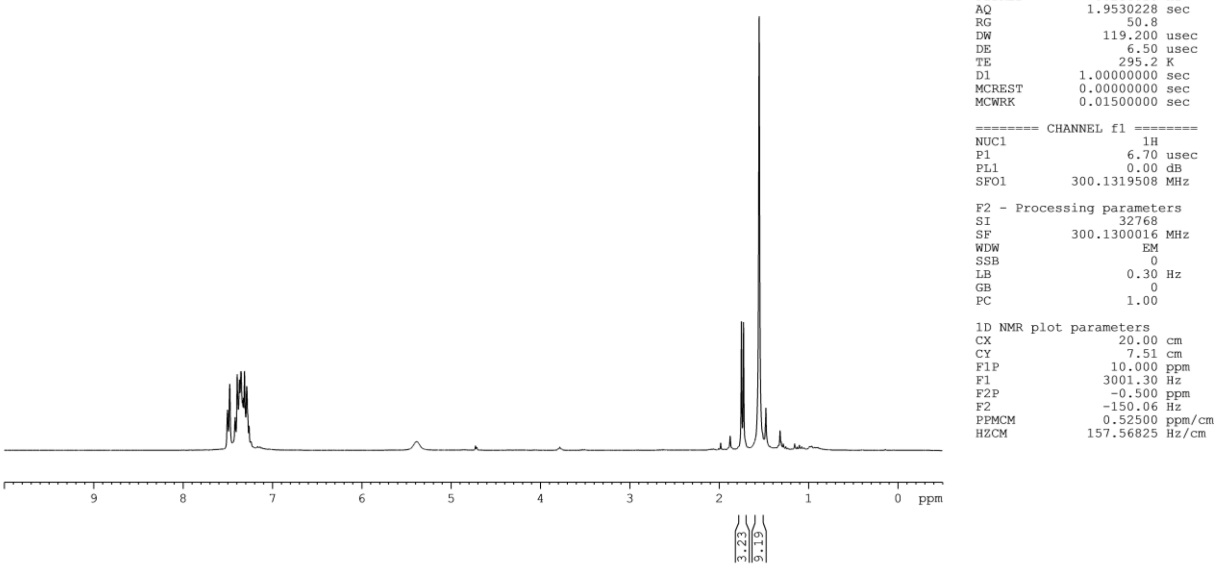
9 8 7 6 5 4 3 2 1 0 ppm
3.23
9.19
Current Data Parameters
NAME fc46wa49bis
EXPNO 2
PROCNO 1
F2 - Acquisition Parameters
Date_ 20121113
Time 18.20
INSTRUM spect
PROBHD 5 mm BBI 1H-BB
PULPROG zg30
TD 16384
SOLVENT CDCl3
NS 16
DS 2
SWH 4194.631 Hz
FIDRES 0.256020 Hz
AQ 1.9530228 sec
RG 50.8
DW 119.200 usec
DE 6.50 usec
TE 295.2 K
D1 1.00000000 sec
MCREST 0.00000000 sec
MCWRK 0.01500000 sec
======== CHANNEL f1 ========
NUC1 1H
P1 6.70 usec
PL1 0.00 dB
SFO1 300.1319508 MHz
F2 - Processing parameters
SI 32768
SF 300.1300016 MHz
WDW EM
SSB 0
LB 0.30 Hz
GB 0
PC 1.00
1D NMR plot parameters
CX 20.00 cm
CY 7.51 cm
F1P 10.000 ppm
F1 3001.30 Hz
F2P -0.500 ppm
F2 -150.06 Hz
PPMCM 0.52500 ppm/cm
HZCM 157.56825 Hz/cm

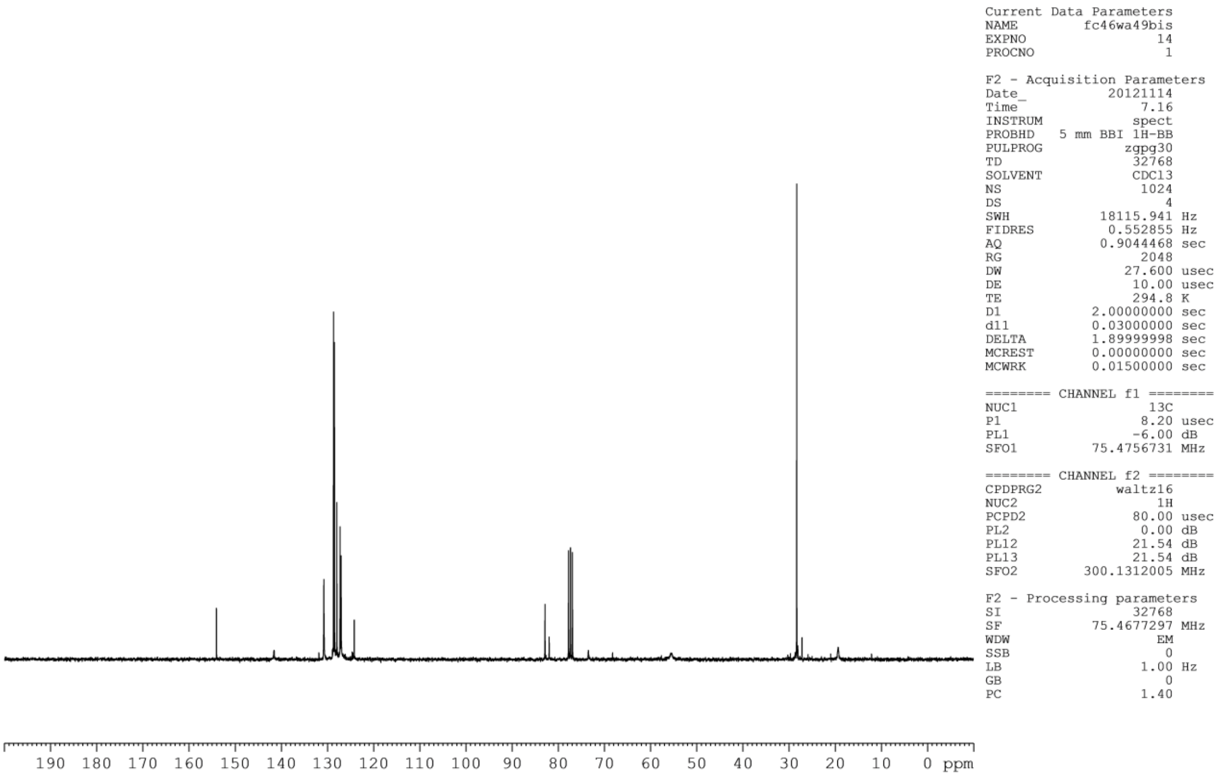
190 180 170 160 150 140 130 120 110 100 90 80 70 60 50 40 30 20 10 0 ppm
Current Data Parameters
NAME fc46wa49bis
EXPNO 14
PROCNO 1
F2 - Acquisition Parameters
Date_ 20121114
Time 7.16
INSTRUM spect
PROBHD 5 mm BBI 1H-BB
PULPROG zgpg30
TD 32768
SOLVENT CDCl3
NS 1024
DS 4
SWH 18115.941 Hz
FIDRES 0.552855 Hz
AQ 0.9044468 sec
RG 2048
DW 27.600 usec
DE 10.00 usec
TE 294.8 K
D1 2.00000000 sec
d11 0.03000000 sec
DELTA 1.89999998 sec
MCREST 0.00000000 sec
MCWRK 0.01500000 sec
======== CHANNEL f1 ========
NUC1 13C
P1 8.20 usec
PL1 -6.00 dB
SFO1 75.4756731 MHz
======== CHANNEL f2 ========
CPDPRG2 waltz16
NUC2 1H
PCPD2 80.00 usec
PL2 0.00 dB
PL12 21.54 dB
PL13 21.54 dB
SFO2 300.1312005 MHz
F2 - Processing parameters
SI 32768
SF 75.4677297 MHz
WDW EM
SSB 0
LB 1.00 Hz
GB 0
PC 1.40

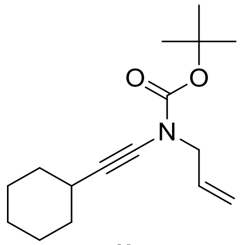

1i

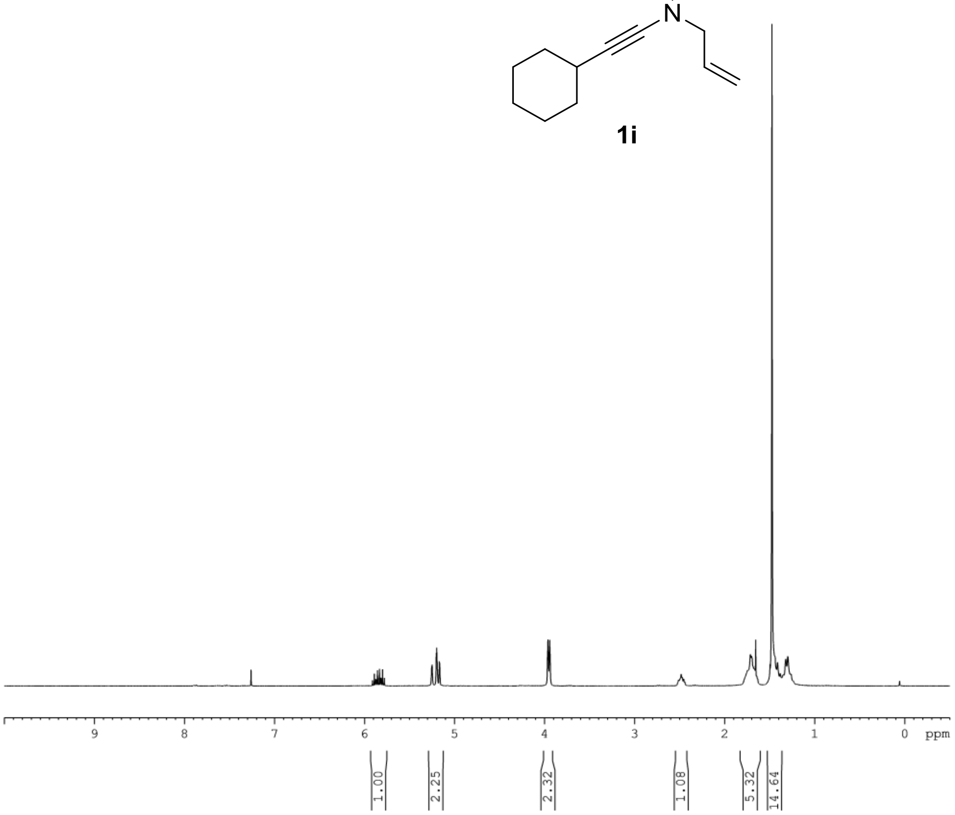


```
Current Data Parameters
NAME             FCWA130
EXPNO                 10
PROCNO                 1

F2 - Acquisition Parameters
Date_           20110514
Time                3.58
INSTRUM            spect
PROBHD   5 mm BBI 1H-BB
PULPROG             zg30
TD                 32768
SOLVENT             C6D6
NS                    16
DS                     0
SWH             6313.131 Hz
FIDRES          0.192661 Hz
AQ             2.5952756 sec
RG                  80.6
DW                79.200 usec
DE                  6.50 usec
TE                 293.8 K
D1            1.00000000 sec
MCREST        0.00000000 sec
MCWRK         0.01500000 sec

======== CHANNEL f1 ========
NUC1                  1H
P1                  6.20 usec
PL1                 0.00 dB
SFO1         300.1328512 MHz

F2 - Processing parameters
SI                 32768
SF           300.1300180 MHz
WDW                   EM
SSB                    0
LB                  0.10 Hz
GB                     0
PC                  1.00

1D NMR plot parameters
CX                 20.00 cm
CY                149.34 cm
F1P                3.500 ppm
F1               1050.45 Hz
F2P               -0.500 ppm
F2               -150.07 Hz
PPMCM            0.20000 ppm/cm
HZCM            60.02600 Hz/cm
```

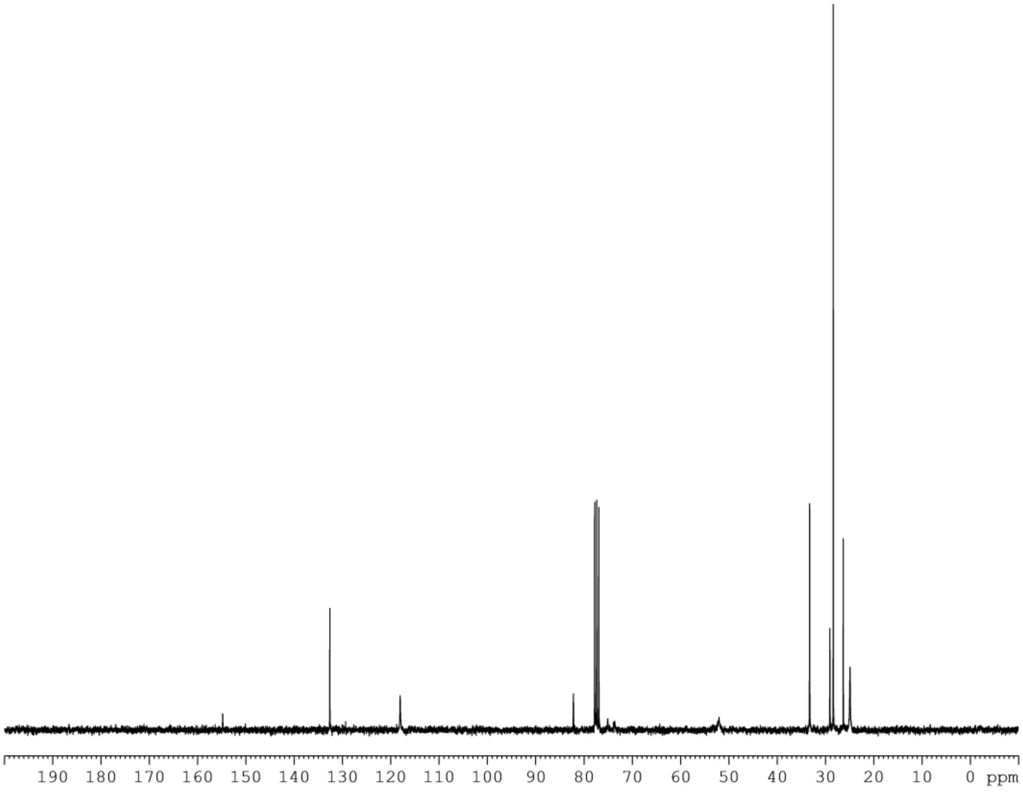


```
Current Data Parameters
NAME             FCWA130
EXPNO                 11
PROCNO                 1

F2 - Acquisition Parameters
Date_           20110514
Time                5.07
INSTRUM            spect
PROBHD   5 mm BBI 1H-BB
PULPROG           zgpg30
TD                 65536
SOLVENT             C6D6
NS                  1024
DS                     4
SWH            17985.611 Hz
FIDRES          0.274439 Hz
AQ             1.8219508 sec
RG                1625.5
DW                27.800 usec
DE                 10.00 usec
TE                 294.0 K
D1            2.00000000 sec
d11           0.03000000 sec
DELTA         1.89999998 sec
MCREST        0.00000000 sec
MCWRK         0.01500000 sec

======== CHANNEL f1 ========
NUC1                 13C
P1                  9.00 usec
PL1                -6.00 dB
SFO1          75.4752953 MHz

======== CHANNEL f2 ========
CPDPRG2          waltz16
NUC2                  1H
PCPD2              80.00 usec
PL2                 0.00 dB
PL12               22.00 dB
PL13               22.00 dB
SFO2         300.1312005 MHz

F2 - Processing parameters
SI                 32768
SF            75.4677452 MHz
WDW                   EM
SSB                    0
LB                  1.00 Hz
GB                     0
PC                  1.40
```

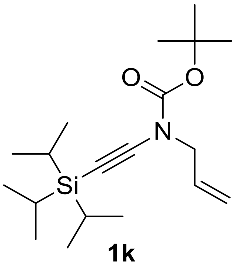
O
O
N
Si

1k

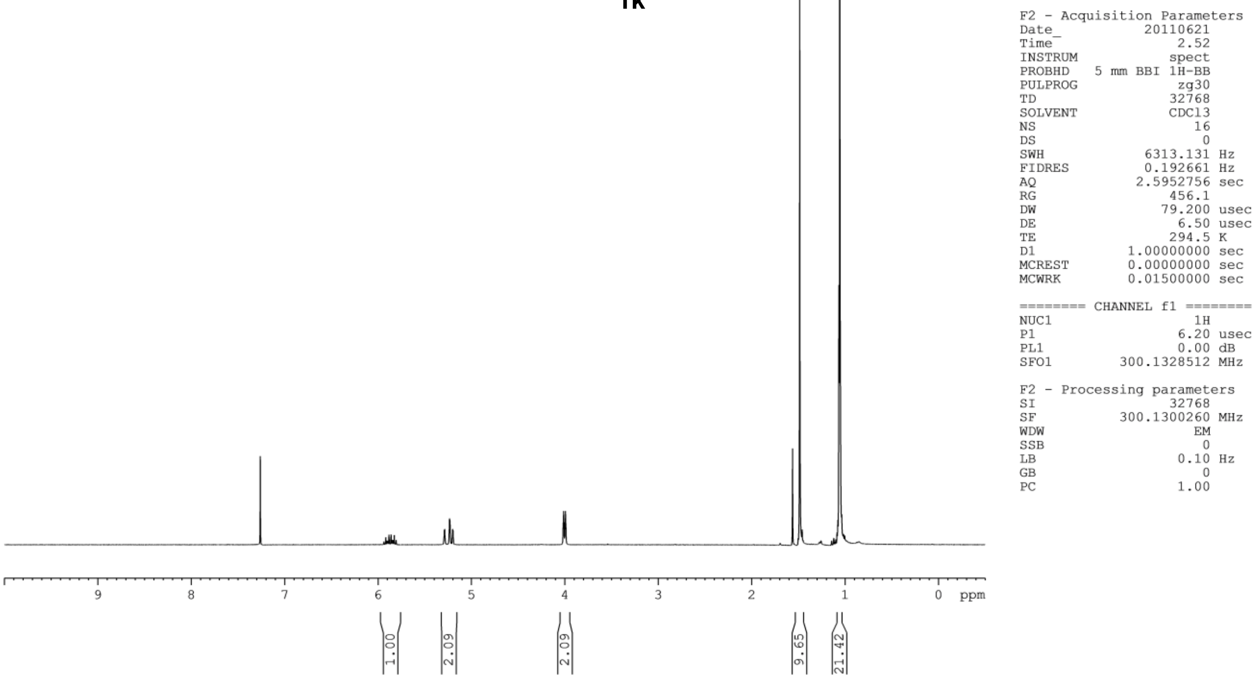
Current Data Parameters
NAME fc25wa170
EXPNO 12
PROCNO 1
F2 - Acquisition Parameters
Date_ 20110621
Time 2.52
INSTRUM spect
PROBHD 5 mm BBI 1H-BB
PULPROG zg30
TD 32768
SOLVENT CDCl3
NS 16
DS 0
SWH 6313.131 Hz
FIDRES 0.192661 Hz
AQ 2.5952756 sec
RG 456.1
DW 79.200 usec
DE 6.50 usec
TE 294.5 K
D1 1.00000000 sec
MCREST 0.00000000 sec
MCWRK 0.01500000 sec
======== CHANNEL f1 ========
NUC1 1H
P1 6.20 usec
PL1 0.00 dB
SFO1 300.1328512 MHz
F2 - Processing parameters
SI 32768
SF 300.1300260 MHz
WDW EM
SSB 0
LB 0.10 Hz
GB 0
PC 1.00
9 8 7 6 5 4 3 2 1 0 ppm
1.00
2.09
2.09
9.65
21.42

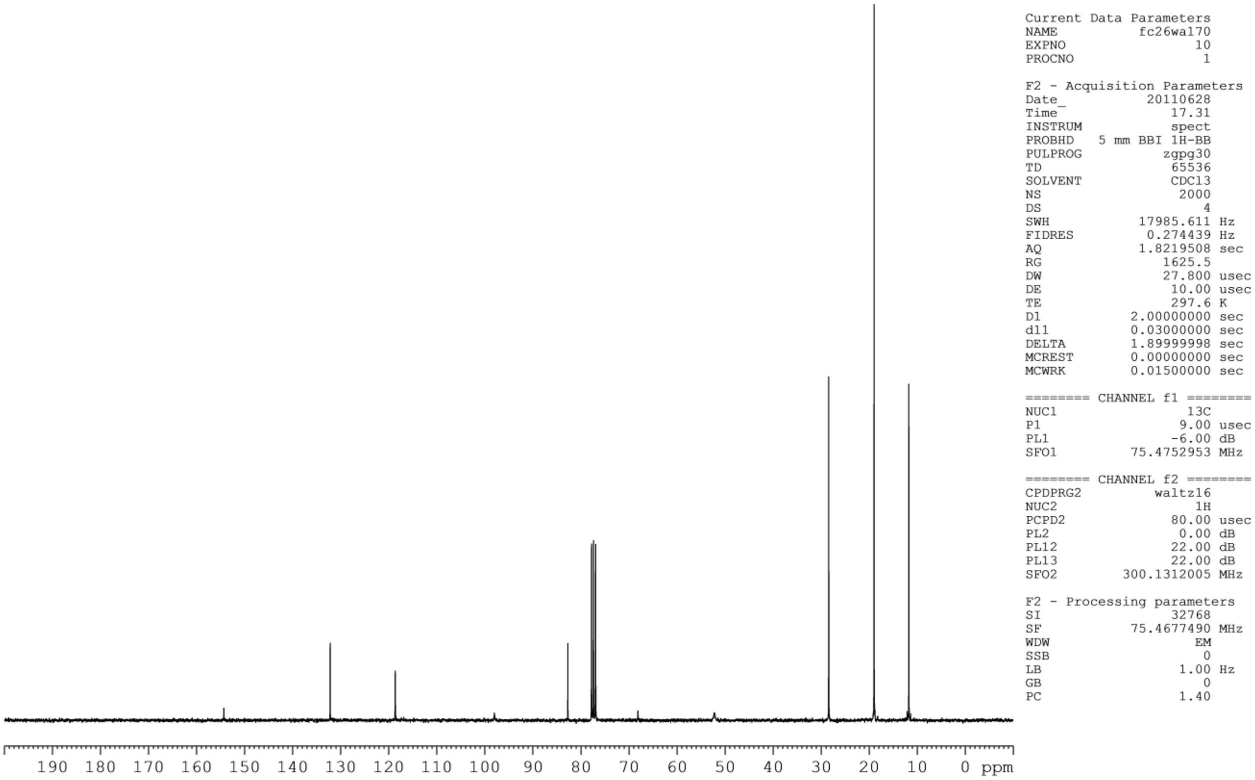
Current Data Parameters
NAME fc26wa170
EXPNO 10
PROCNO 1
F2 - Acquisition Parameters
Date_ 20110628
Time 17.31
INSTRUM spect
PROBHD 5 mm BBI 1H-BB
PULPROG zgpg30
TD 65536
SOLVENT CDCl3
NS 2000
DS 4
SWH 17985.611 Hz
FIDRES 0.274439 Hz
AQ 1.8219508 sec
RG 1625.5
DW 27.800 usec
DE 10.00 usec
TE 297.6 K
D1 2.00000000 sec
d11 0.03000000 sec
DELTA 1.89999998 sec
MCREST 0.00000000 sec
MCWRK 0.01500000 sec
======== CHANNEL f1 ========
NUC1 13C
P1 9.00 usec
PL1 -6.00 dB
SFO1 75.4752953 MHz
======== CHANNEL f2 ========
CPDPRG2 waltz16
NUC2 1H
PCPD2 80.00 usec
PL2 0.00 dB
PL12 22.00 dB
PL13 22.00 dB
SFO2 300.1312005 MHz
F2 - Processing parameters
SI 32768
SF 75.4677490 MHz
WDW EM
SSB 0
LB 1.00 Hz
GB 0
PC 1.40
190 180 170 160 150 140 130 120 110 100 90 80 70 60 50 40 30 20 10 0 ppm

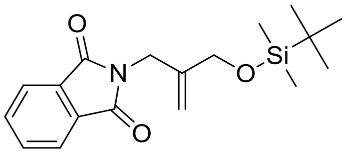


```
Current Data Parameters
NAME             fc41wa511
EXPNO                   10
PROCNO                   1

F2 - Acquisition Parameters
Date_             20121014
Time                  4.03
INSTRUM              spect
PROBHD   5 mm BBI 1H-BB
PULPROG               zg30
TD                   16384
SOLVENT              CDCl3
NS                      16
DS                       2
SWH               4194.631 Hz
FIDRES            0.256020 Hz
AQ               1.9530228 sec
RG                   287.4
DW                 119.200 usec
DE                    6.50 usec
TE                   292.4 K
D1              1.00000000 sec
MCREST          0.00000000 sec
MCWRK           0.01500000 sec

======== CHANNEL f1 ========
NUC1                    1H
P1                    6.70 usec
PL1                   0.00 dB
SFO1           300.1319508 MHz

F2 - Processing parameters
SI                   32768
SF             300.1300209 MHz
WDW                     EM
SSB                      0
LB                    0.30 Hz
GB                       0
PC                    1.00
```

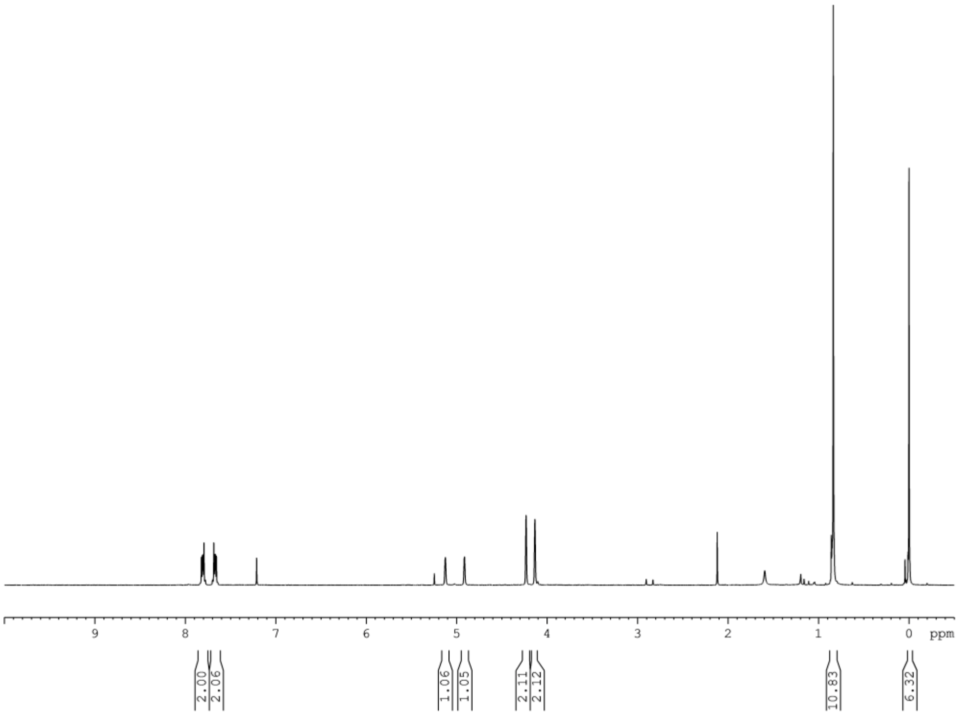


```
Current Data Parameters
NAME             fc41wa511
EXPNO                   12
PROCNO                   1

F2 - Acquisition Parameters
Date_             20121014
Time                  5.09
INSTRUM              spect
PROBHD   5 mm BBI 1H-BB
PULPROG             zgpg30
TD                   32768
SOLVENT              CDCl3
NS                    1024
DS                       4
SWH              18115.941 Hz
FIDRES            0.552855 Hz
AQ               0.9044468 sec
RG                    2048
DW                  27.600 usec
DE                   10.00 usec
TE                   292.4 K
D1              2.00000000 sec
d11             0.03000000 sec
DELTA           1.89999998 sec
MCREST          0.00000000 sec
MCWRK           0.01500000 sec

======== CHANNEL f1 ========
NUC1                   13C
P1                    8.20 usec
PL1                  -6.00 dB
SFO1            75.4756731 MHz

======== CHANNEL f2 ========
CPDPRG2            waltz16
NUC2                    1H
PCPD2                80.00 usec
PL2                   0.00 dB
PL12                 21.54 dB
PL13                 21.54 dB
SFO2           300.1312005 MHz

F2 - Processing parameters
SI                   32768
SF              75.4677490 MHz
WDW                     EM
SSB                      0
LB                    1.00 Hz
GB                       0
PC                    1.40
```

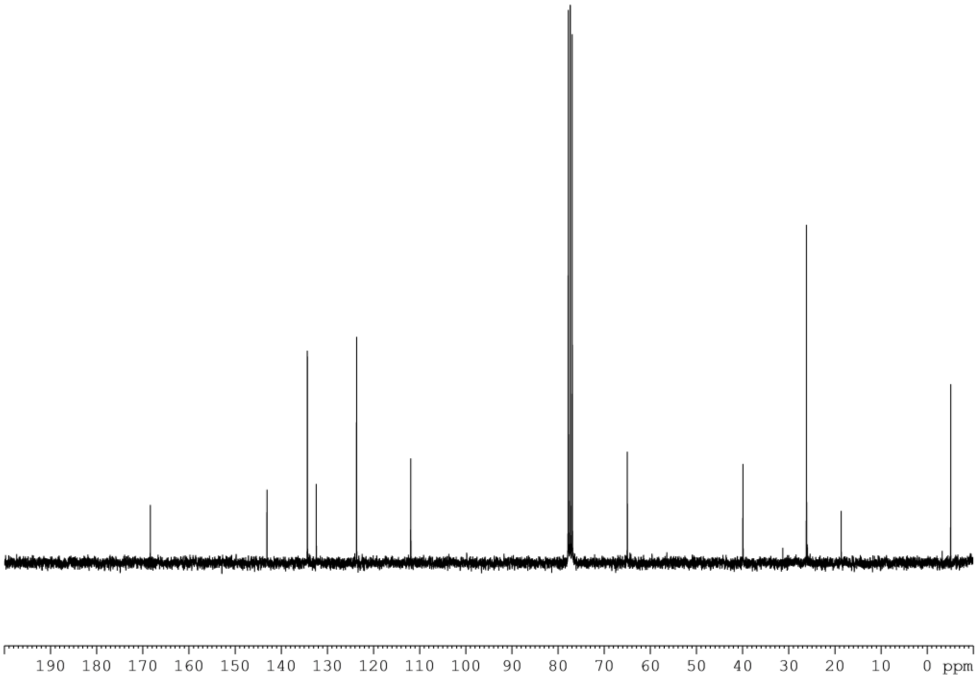

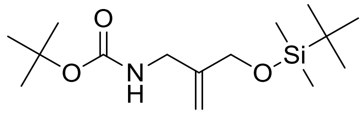

13

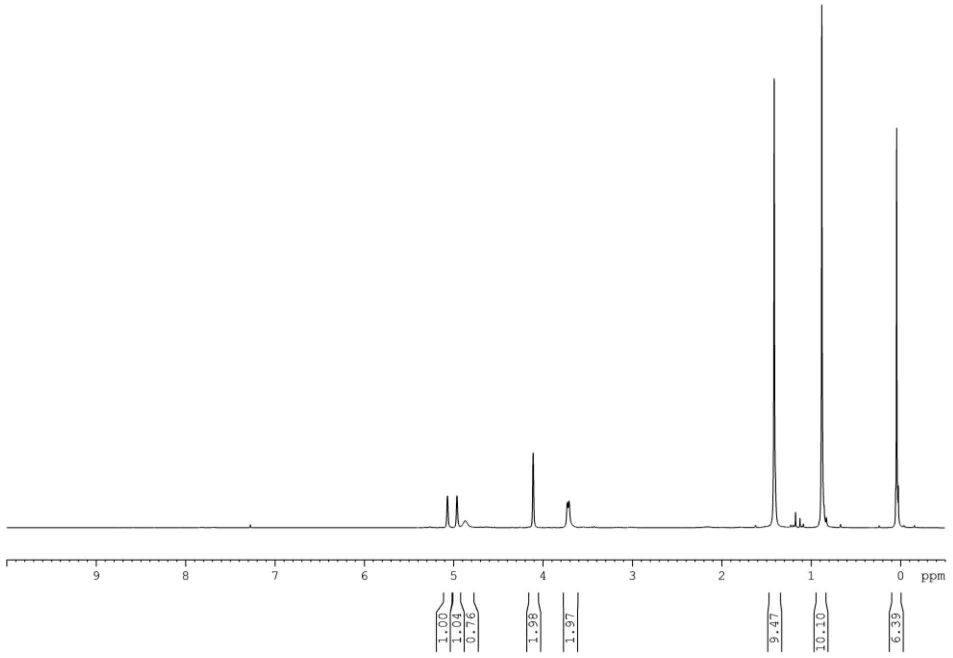


```
Current Data Parameters
NAME         fc41wa509-1
EXPNO                  1
PROCNO                 1

F2 - Acquisition Parameters
Date_           20121011
Time               10.49
INSTRUM            spect
PROBHD   5 mm BBI 1H-BB
PULPROG             zg30
TD                 16384
SOLVENT            CDCl3
NS                    16
DS                     2
SWH             4194.631 Hz
FIDRES          0.256020 Hz
AQ             1.9530228 sec
RG                  35.9
DW               119.200 usec
DE                  6.50 usec
TE                 293.6 K
D1            1.00000000 sec
MCREST        0.00000000 sec
MCWRK         0.01500000 sec

======== CHANNEL f1 ========
NUC1                  1H
P1                  6.70 usec
PL1                 0.00 dB
SFO1         300.1319508 MHz

F2 - Processing parameters
SI                 32768
SF           300.1300016 MHz
WDW                   EM
SSB                    0
LB                  0.30 Hz
GB                     0
PC                  1.00

1D NMR plot parameters
CX                 20.00 cm
CY                 10.00 cm
F1P               10.000 ppm
F1               3001.30 Hz
F2P               -0.500 ppm
F2               -150.06 Hz
PPMCM            0.52500 ppm/cm
HZCM           157.56825 Hz/cm
```

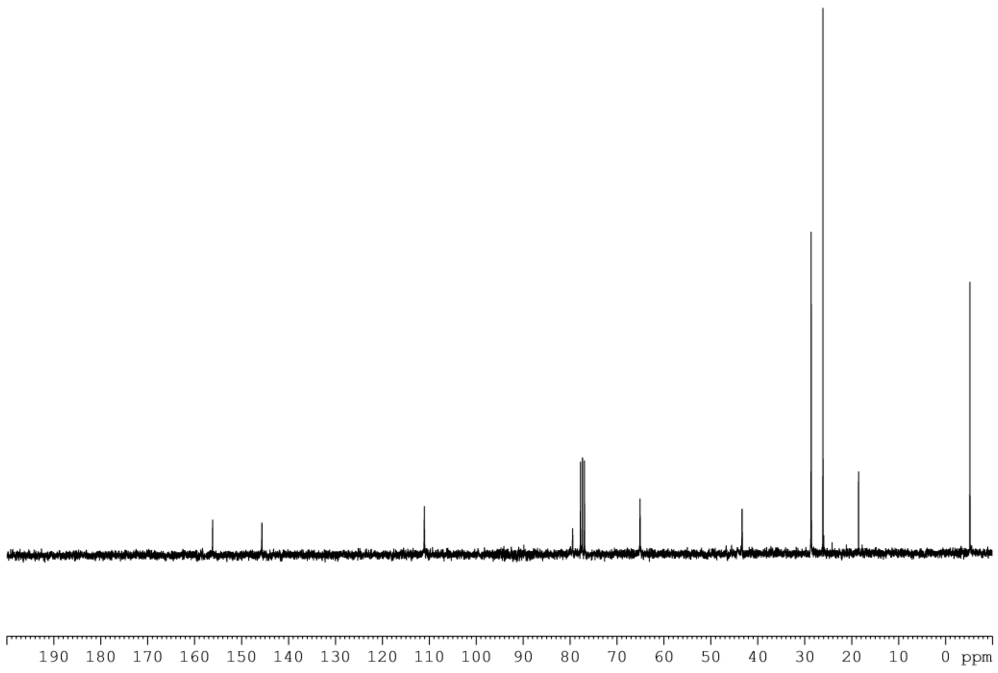


```
Current Data Parameters
NAME         fc41wa509-1
EXPNO                  2
PROCNO                 1

F2 - Acquisition Parameters
Date_           20121011
Time               10.53
INSTRUM            spect
PROBHD   5 mm BBI 1H-BB
PULPROG           zgpg30
TD                 32768
SOLVENT            CDCl3
NS                    55
DS                     4
SWH            18115.941 Hz
FIDRES          0.552855 Hz
AQ             0.9044468 sec
RG                  2048
DW                27.600 usec
DE                 10.00 usec
TE                 293.6 K
D1            2.00000000 sec
d11           0.03000000 sec
DELTA         1.89999998 sec
MCREST        0.00000000 sec
MCWRK         0.01500000 sec

======== CHANNEL f1 ========
NUC1                 13C
P1                  8.20 usec
PL1                -6.00 dB
SFO1          75.4756731 MHz

======== CHANNEL f2 ========
CPDPRG2          waltz16
NUC2                  1H
PCPD2              80.00 usec
PL2                 0.00 dB
PL12               21.54 dB
PL13               21.54 dB
SFO2         300.1312005 MHz

F2 - Processing parameters
SI                 32768
SF            75.4677490 MHz
WDW                   EM
SSB                    0
LB                  1.00 Hz
GB                     0
PC                  1.40

1D NMR plot parameters
CX                 20.00 cm
CY                  9.41 cm
F1P              200.000 ppm
F1              15093.55 Hz
F2P              -10.000 ppm
F2               -754.68 Hz
PPMCM           10.50000 ppm/cm
HZCM           792.41138 Hz/cm
```

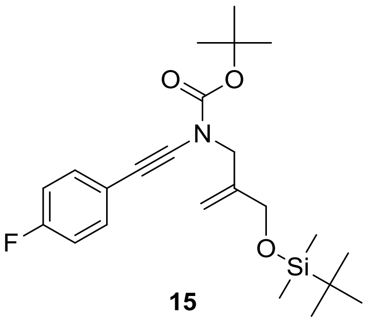

15

```
Current Data Parameters
NAME           fc41wa512
EXPNO                 10
PROCNO                 1

F2 - Acquisition Parameters
Date_           20121014
Time               17.24
INSTRUM            spect
PROBHD   5 mm BBI 1H-BB
PULPROG             zg30
TD                 16384
SOLVENT            CDCl3
NS                    16
DS                     2
SWH             4194.631 Hz
FIDRES          0.256020 Hz
AQ             1.9530228 sec
RG                    64
DW               119.200 usec
DE                  6.50 usec
TE                 292.0 K
D1            1.00000000 sec
MCREST        0.00000000 sec
MCWRK         0.01500000 sec

======== CHANNEL f1 ========
NUC1                  1H
P1                  6.70 usec
PL1                 0.00 dB
SFO1         300.1319508 MHz

F2 - Processing parameters
SI                 32768
SF           300.1300268 MHz
WDW                   EM
SSB                    0
LB                  0.30 Hz
GB                     0
PC                  1.00
```

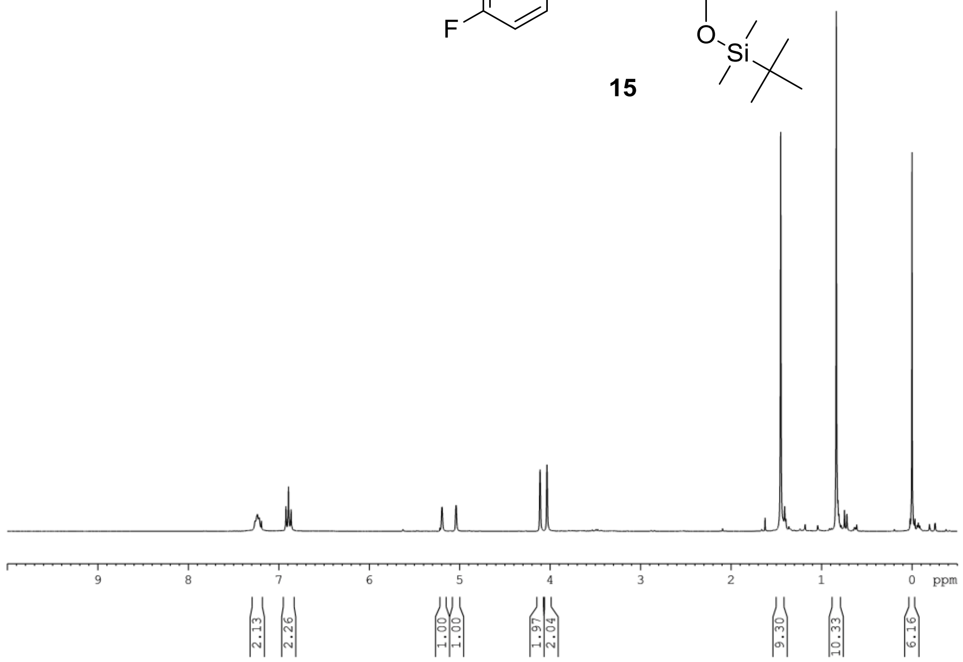


```
Current Data Parameters
NAME           fc41wa512
EXPNO                 14
PROCNO                 1

F2 - Acquisition Parameters
Date_           20121014
Time               21.54
INSTRUM            spect
PROBHD   5 mm BBI 1H-BB
PULPROG           zgpg30
TD                 32768
SOLVENT            CDCl3
NS                  2000
DS                     4
SWH            18115.941 Hz
FIDRES          0.552855 Hz
AQ             0.9044468 sec
RG                  2048
DW                27.600 usec
DE                 10.00 usec
TE                 292.1 K
D1            4.00000000 sec
d11           0.03000000 sec
DELTA         3.90000010 sec
MCREST        0.00000000 sec
MCWRK         0.01500000 sec

======== CHANNEL f1 ========
NUC1                 13C
P1                  8.20 usec
PL1                -6.00 dB
SFO1          75.4756731 MHz

======== CHANNEL f2 ========
CPDPRG2          waltz16
NUC2                  1H
PCPD2              80.00 usec
PL2                 0.00 dB
PL12               21.54 dB
PL13               21.54 dB
SFO2         300.1312005 MHz

F2 - Processing parameters
SI                 32768
SF            75.4677490 MHz
WDW                   EM
SSB                    0
LB                  1.00 Hz
GB                     0
PC                  1.40
```

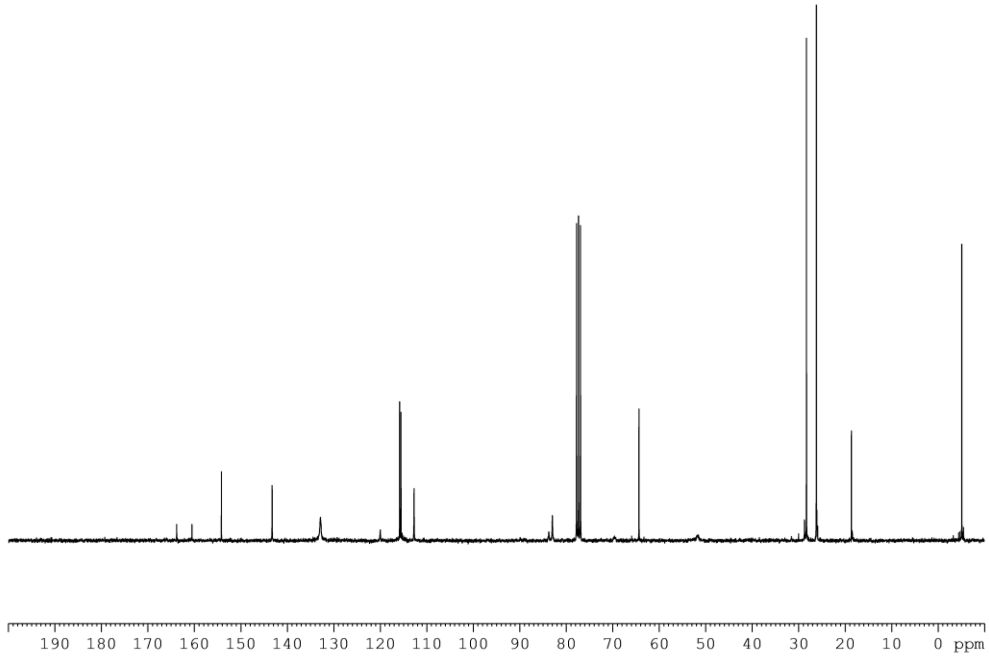

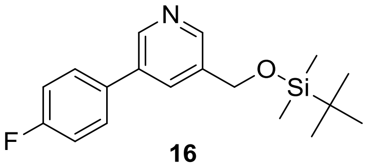

16

```
Current Data Parameters
NAME          fc44wa535
EXPNO                 1
PROCNO                1

F2 - Acquisition Parameters
Date_          20121030
Time              15.21
INSTRUM           spect
PROBHD   5 mm BBI 1H-BB
PULPROG            zg30
TD                16384
SOLVENT           CDCl3
NS                   16
DS                    2
SWH            4194.631 Hz
FIDRES         0.256020 Hz
AQ            1.9530228 sec
RG                   57
DW              119.200 usec
DE                 6.50 usec
TE                295.0 K
D1           1.00000000 sec
MCREST       0.00000000 sec
MCWRK        0.01500000 sec

======== CHANNEL f1 ========
NUC1                 1H
P1                 6.70 usec
PL1                0.00 dB
SFO1        300.1319508 MHz

F2 - Processing parameters
SI                32768
SF          300.1300016 MHz
WDW                  EM
SSB                   0
LB                 0.30 Hz
GB                    0
PC                 1.00

1D NMR plot parameters
CX                20.00 cm
CY                 9.55 cm
F1P              10.000 ppm
F1              3001.30 Hz
F2P              -0.500 ppm
F2              -150.06 Hz
PPMCM           0.52500 ppm/cm
HZCM          157.56825 Hz/cm
```

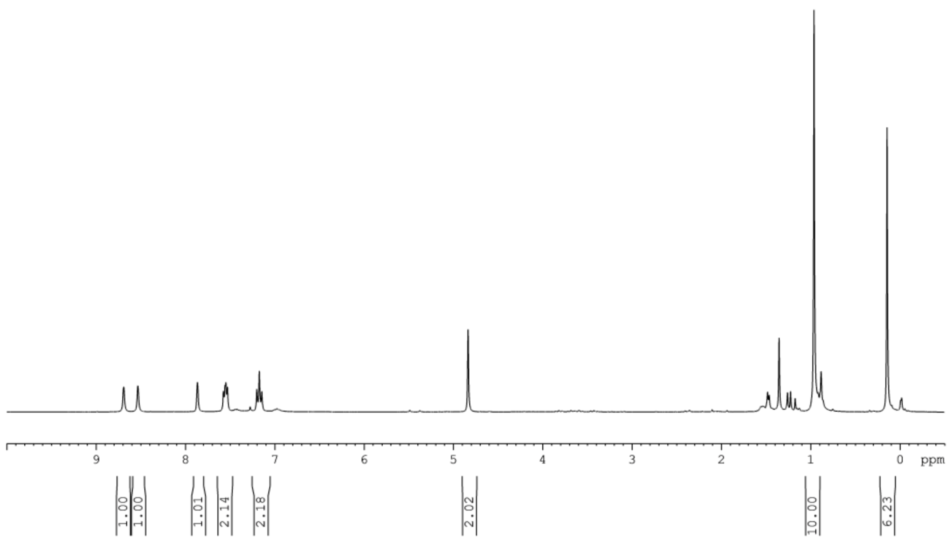


```
Current Data Parameters
NAME          fc44wa535
EXPNO                 2
PROCNO                1

F2 - Acquisition Parameters
Date_          20121030
Time              15.25
INSTRUM           spect
PROBHD   5 mm BBI 1H-BB
PULPROG          zgpg30
TD                32768
SOLVENT           CDCl3
NS                  100
DS                    4
SWH           18115.941 Hz
FIDRES         0.552855 Hz
AQ            0.9044468 sec
RG                 2048
DW               27.600 usec
DE                10.00 usec
TE                295.1 K
D1           2.00000000 sec
d11          0.03000000 sec
DELTA        1.89999998 sec
MCREST       0.00000000 sec
MCWRK        0.01500000 sec

======== CHANNEL f1 ========
NUC1                13C
P1                 8.20 usec
PL1               -6.00 dB
SFO1         75.4756731 MHz

======== CHANNEL f2 ========
CPDPRG2         waltz16
NUC2                 1H
PCPD2             80.00 usec
PL2                0.00 dB
PL12              21.54 dB
PL13              21.54 dB
SFO2        300.1312005 MHz

F2 - Processing parameters
SI                32768
SF           75.4677490 MHz
WDW                  EM
SSB                   0
LB                 1.00 Hz
GB                    0
PC                 1.40

1D NMR plot parameters
CX                20.00 cm
CY                 6.89 cm
F1P             200.000 ppm
F1             15093.55 Hz
F2P             -10.000 ppm
F2              -754.68 Hz
PPMCM          10.50000 ppm/cm
HZCM          792.41138 Hz/cm
```

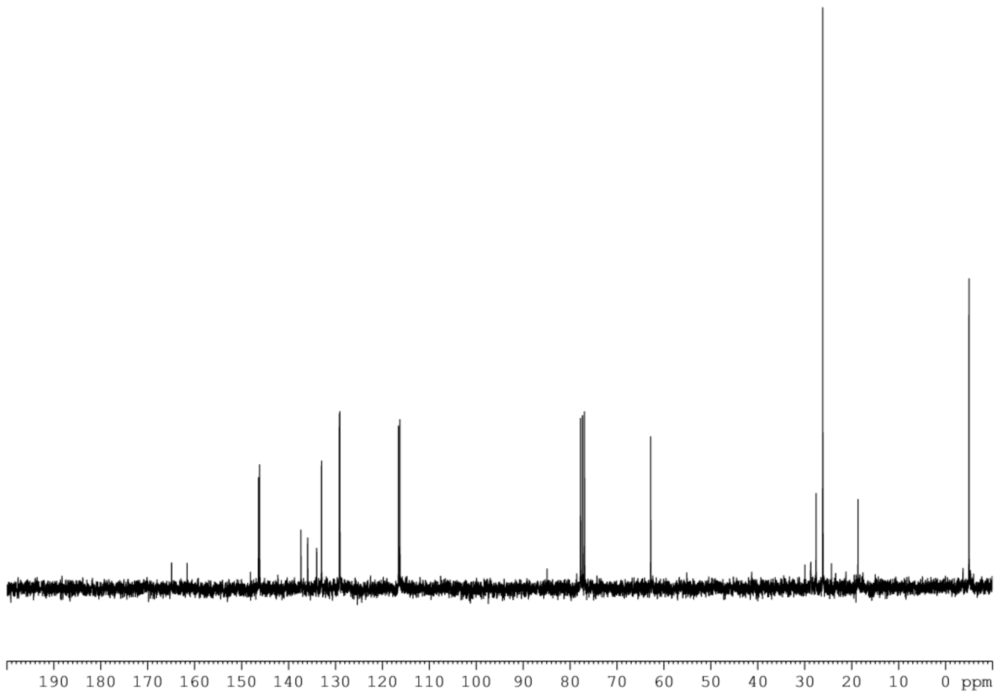

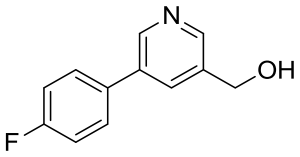
N
OH
F

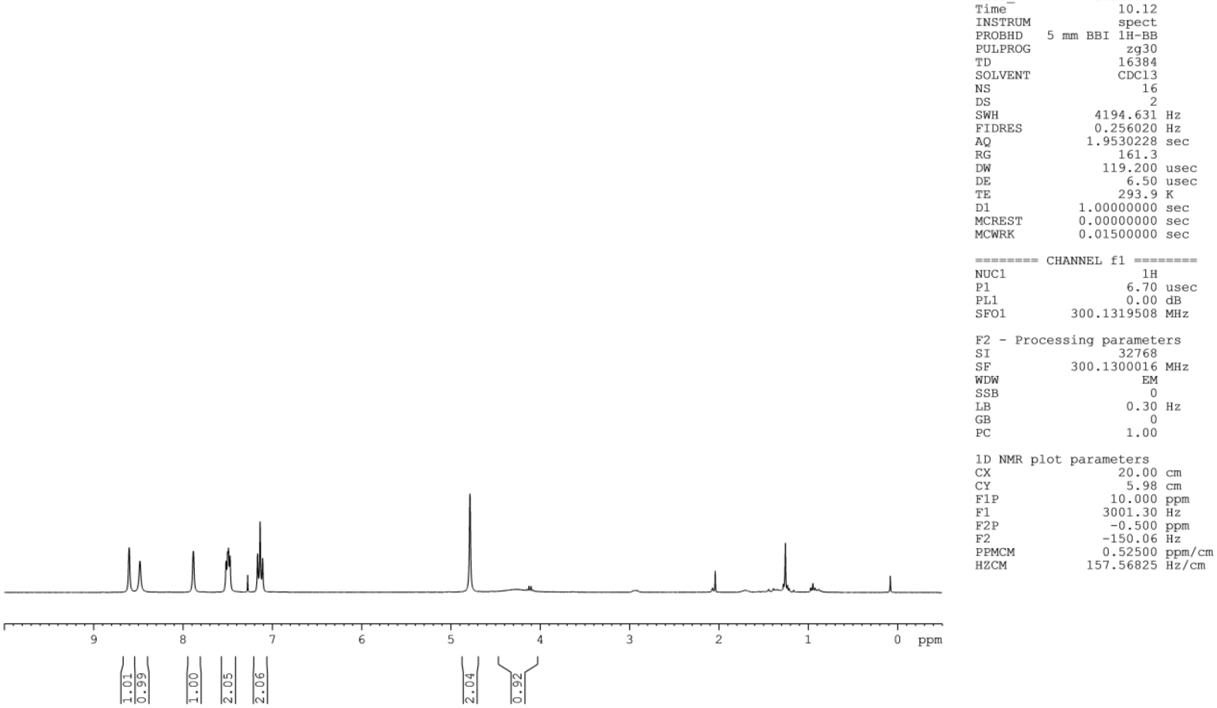
Current Data Parameters
NAME fc45wa539
EXPNO 1
PROCNO 1
F2 - Acquisition Parameters
Date_ 20121106
Time 10.12
INSTRUM spect
PROBHD 5 mm BBI 1H-BB
PULPROG zg30
TD 16384
SOLVENT CDCl3
NS 16
DS 2
SWH 4194.631 Hz
FIDRES 0.256020 Hz
AQ 1.9530228 sec
RG 161.3
DW 119.200 usec
DE 6.50 usec
TE 293.9 K
D1 1.00000000 sec
MCREST 0.00000000 sec
MCWRK 0.01500000 sec
======== CHANNEL f1 ========
NUC1 1H
P1 6.70 usec
PL1 0.00 dB
SFO1 300.1319508 MHz
F2 - Processing parameters
SI 32768
SF 300.1300016 MHz
WDW EM
SSB 0
LB 0.30 Hz
GB 0
PC 1.00
1D NMR plot parameters
CX 20.00 cm
CY 5.98 cm
F1P 10.000 ppm
F1 3001.30 Hz
F2P -0.500 ppm
F2 -150.06 Hz
PPMCM 0.52500 ppm/cm
HZCM 157.56825 Hz/cm
9
8
7
6
5
4
3
2
1
0 ppm
1.01
0.99
1.00
2.05
2.06
2.04
0.92

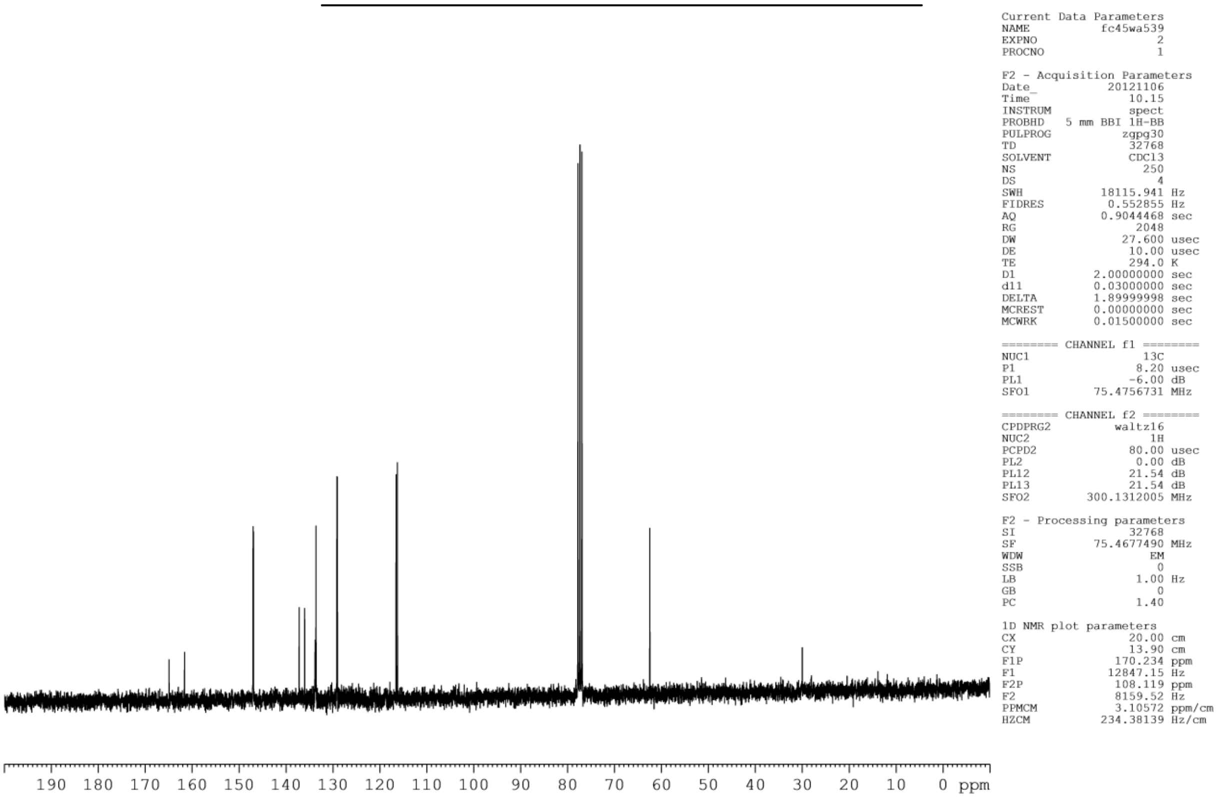
Current Data Parameters
NAME fc45wa539
EXPNO 2
PROCNO 1
F2 - Acquisition Parameters
Date_ 20121106
Time 10.15
INSTRUM spect
PROBHD 5 mm BBI 1H-BB
PULPROG zgpg30
TD 32768
SOLVENT CDCl3
NS 250
DS 4
SWH 18115.941 Hz
FIDRES 0.552855 Hz
AQ 0.9044468 sec
RG 2048
DW 27.600 usec
DE 10.00 usec
TE 294.0 K
D1 2.00000000 sec
d11 0.03000000 sec
DELTA 1.89999998 sec
MCREST 0.00000000 sec
MCWRK 0.01500000 sec
======== CHANNEL f1 ========
NUC1 13C
P1 8.20 usec
PL1 -6.00 dB
SFO1 75.4756731 MHz
======== CHANNEL f2 ========
CPDPRG2 waltz16
NUC2 1H
PCPD2 80.00 usec
PL2 0.00 dB
PL12 21.54 dB
PL13 21.54 dB
SFO2 300.1312005 MHz
F2 - Processing parameters
SI 32768
SF 75.4677490 MHz
WDW EM
SSB 0
LB 1.00 Hz
GB 0
PC 1.40
1D NMR plot parameters
CX 20.00 cm
CY 13.90 cm
F1P 170.234 ppm
F1 12847.15 Hz
F2P 108.119 ppm
F2 8159.52 Hz
PPMCM 3.10572 ppm/cm
HZCM 234.38139 Hz/cm
190
180
170
160
150
140
130
120
110
100
90
80
70
60
50
40
30
20
10
0 ppm

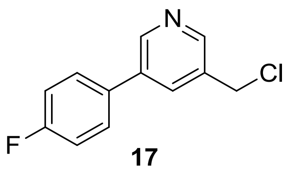
N
Cl
F

17

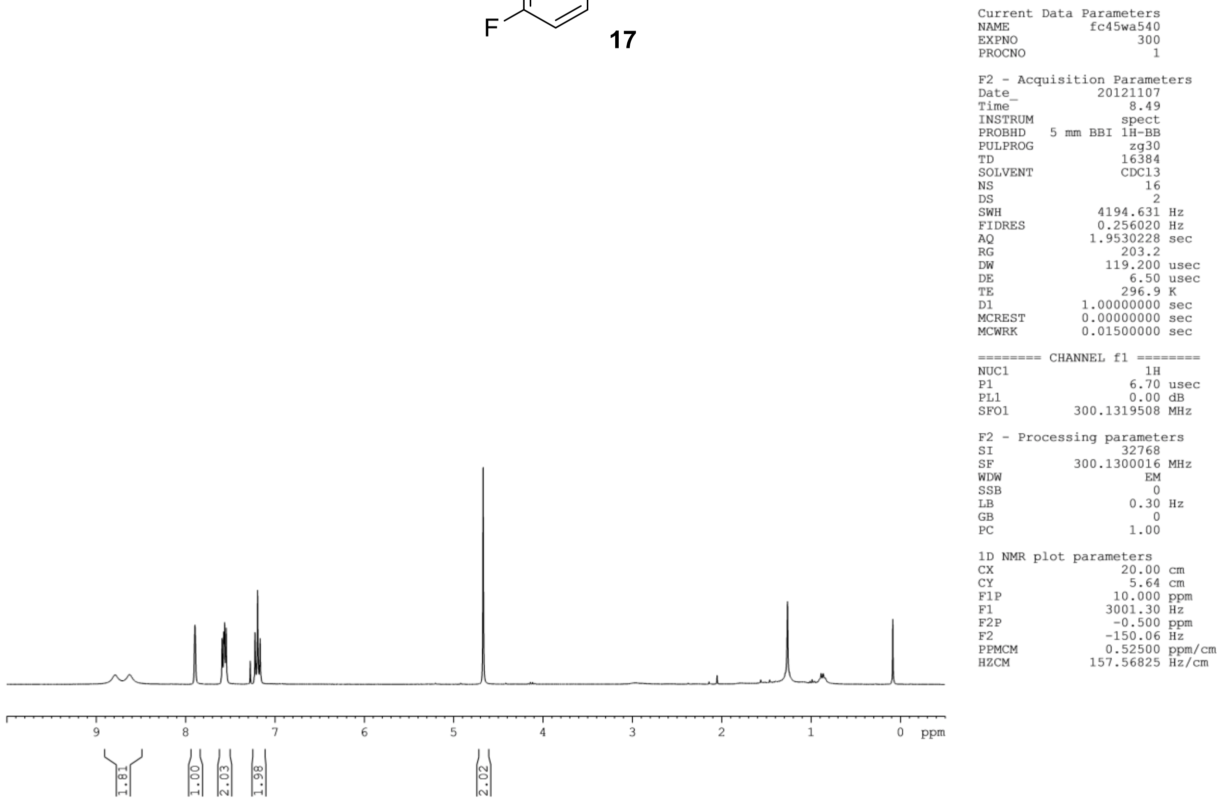
Current Data Parameters
NAME fc45wa540
EXPNO 300
PROCNO 1
F2 - Acquisition Parameters
Date_ 20121107
Time 8.49
INSTRUM spect
PROBHD 5 mm BBI 1H-BB
PULPROG zg30
TD 16384
SOLVENT CDCl3
NS 16
DS 2
SWH 4194.631 Hz
FIDRES 0.256020 Hz
AQ 1.9530228 sec
RG 203.2
DW 119.200 usec
DE 6.50 usec
TE 296.9 K
D1 1.00000000 sec
MCREST 0.00000000 sec
MCWRK 0.01500000 sec
======== CHANNEL f1 ========
NUC1 1H
P1 6.70 usec
PL1 0.00 dB
SFO1 300.1319508 MHz
F2 - Processing parameters
SI 32768
SF 300.1300016 MHz
WDW EM
SSB 0
LB 0.30 Hz
GB 0
PC 1.00
1D NMR plot parameters
CX 20.00 cm
CY 5.64 cm
F1P 10.000 ppm
F1 3001.30 Hz
F2P -0.500 ppm
F2 -150.06 Hz
PPMCM 0.52500 ppm/cm
HZCM 157.56825 Hz/cm
9 8 7 6 5 4 3 2 1 0 ppm
1.81
1.00
2.03
1.98
2.02

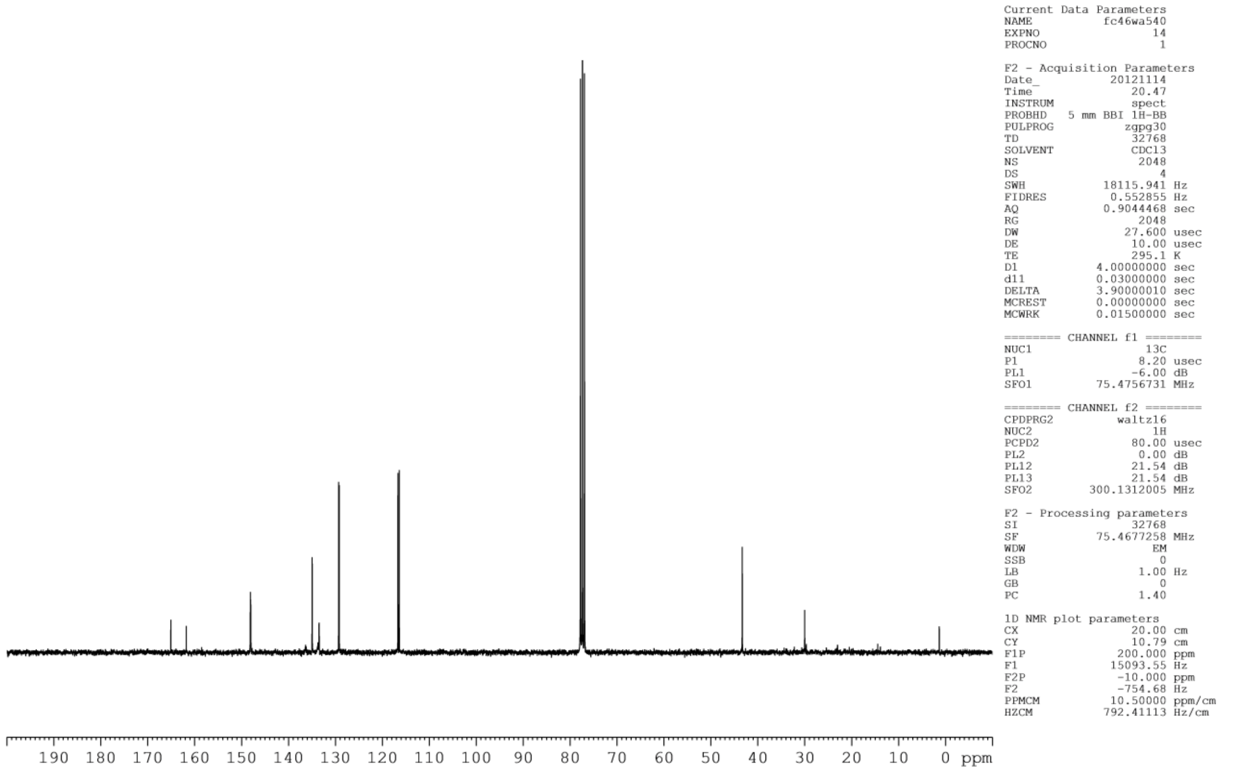
Current Data Parameters
NAME fc46wa540
EXPNO 14
PROCNO 1
F2 - Acquisition Parameters
Date_ 20121114
Time 20.47
INSTRUM spect
PROBHD 5 mm BBI 1H-BB
PULPROG zgpg30
TD 32768
SOLVENT CDCl3
NS 2048
DS 4
SWH 18115.941 Hz
FIDRES 0.552855 Hz
AQ 0.9044468 sec
RG 2048
DW 27.600 usec
DE 10.00 usec
TE 295.1 K
D1 4.00000000 sec
d11 0.03000000 sec
DELTA 3.90000010 sec
MCREST 0.00000000 sec
MCWRK 0.01500000 sec
======== CHANNEL f1 ========
NUC1 13C
P1 8.20 usec
PL1 -6.00 dB
SFO1 75.4756731 MHz
======== CHANNEL f2 ========
CPDPRG2 waltz16
NUC2 1H
PCPD2 80.00 usec
PL2 0.00 dB
PL12 21.54 dB
PL13 21.54 dB
SFO2 300.1312005 MHz
F2 - Processing parameters
SI 32768
SF 75.4677258 MHz
WDW EM
SSB 0
LB 1.00 Hz
GB 0
PC 1.40
1D NMR plot parameters
CX 20.00 cm
CY 10.79 cm
F1P 200.000 ppm
F1 15093.55 Hz
F2P -10.000 ppm
F2 -754.68 Hz
PPMCM 10.50000 ppm/cm
HZCM 792.41113 Hz/cm
190 180 170 160 150 140 130 120 110 100 90 80 70 60 50 40 30 20 10 0 ppm
